# Organic Particles: Heterogeneous Hubs for Microbial Interactions in Aquatic Ecosystems

**DOI:** 10.3389/fmicb.2018.02569

**Published:** 2018-10-26

**Authors:** Mina Bižić-Ionescu, Danny Ionescu, Hans-Peter Grossart

**Affiliations:** ^1^Leibniz-Institute of Freshwater Ecology and Inland Fisheries, Stechlin, Germany; ^2^Institute of Biochemistry and Biology, University of Potsdam, Potsdam, Germany

**Keywords:** particle-associated bacteria, microbial communities, inter- and intra-species interactions, antagonism, phage, transcriptome

## Abstract

The dynamics and activities of microbes colonizing organic particles (hereafter particles) greatly determine the efficiency of the aquatic carbon pump. Current understanding is that particle composition, structure and surface properties, determined mostly by the forming organisms and organic matter, dictate initial microbial colonization and the subsequent rapid succession events taking place as organic matter lability and nutrient content change with microbial degradation. We applied a transcriptomic approach to assess the role of stochastic events on initial microbial colonization of particles. Furthermore, we asked whether gene expression corroborates rapid changes in carbon-quality. Commonly used size fractionated filtration averages thousands of particles of different sizes, sources, and ages. To overcome this drawback, we used replicate samples consisting each of 3–4 particles of identical source and age and further evaluated the consequences of averaging 10–1000s of particles. Using flow-through rolling tanks we conducted long-term experiments at near *in situ* conditions minimizing the biasing effects of closed incubation approaches often referred to as “the bottle-effect.” In our open flow-through rolling tank system, however, active microbial communities were highly heterogeneous despite an identical particle source, suggesting random initial colonization. Contrasting previous reports using closed incubation systems, expression of carbon utilization genes didn’t change after 1 week of incubation. Consequently, we suggest that in nature, changes in particle-associated community related to carbon availability are much slower (days to weeks) due to constant supply of labile, easily degradable organic matter. Initial, random particle colonization seems to be subsequently altered by multiple organismic interactions shaping microbial community interactions and functional dynamics. Comparative analysis of thousands particles pooled togethers as well as pooled samples suggests that mechanistic studies of microbial dynamics should be done on single particles. The observed microbial heterogeneity and inter-organismic interactions may have important implications for evolution and biogeochemistry in aquatic systems.

## Introduction

Organic particles (hereafter particles) and their surrounding solutes plume are hotspots of microbial activity in aquatic systems (Kiørboe, 2001; Simon et al., 2002). They are usually derived from several sources such as live and dead phyto- and zooplankton, fecal-pellets or allochthonous organic matter (Jackson, 1980; Alldredge and Silver, 1988; Grossart and Simon, 1993; Simon et al., 2002). Carbon and nutrients on particles often exceed background levels by 2–4 orders of magnitude (Prézelin and Alldredge, 1983; Grossart and Simon, 1998b) and thus particles are heavily colonized by microorganisms (McCarthy and Goldman, 1979; Shanks and Trent, 1979; Smriga et al., 2016) including heterotrophic bacteria (Alldredge and Youngbluth, 1985; Alldredge et al., 1986) and fungi (Wurzbacher et al., 2014). Consequently, microbial dynamics play an essential role in organic matter degradation (Alldredge and Silver, 1988; Simon et al., 2002a), aggregation and sinking and thus for the biological (De La Rocha and Passow, 2007) and microbial (Jiao and Azam, 2011; Legendre et al., 2015) carbon pump efficiency.

Microorganisms consume and solubilize particles into dissolved organic matter by a suite of ectohydrolases (Smith et al., 1992; Grossart and Simon, 1998a), but not all microorganisms possess the enzymatic machinery for all organic matter types (Kiørboe et al., 2002). Thus, microbial organic matter consumption over time will greatly affect the particle’s carbon quantity and quality, increasing the semi-labile and refractory fractions over the labile, rapidly bioavailable carbon. This will lead to pronounced temporal shifts in particle-associated community composition since initial communities will be outcompeted by more specialized ones (Kiørboe et al., 2003; Datta et al., 2016). Therefore, changes in particle composition will shape dynamics and composition of the associated communities (Grossart, 1999; Eiler and Bertilsson, 2004; Grossart et al., 2005; LeCleir et al., 2014). In addition, particle associated communities are rarely homogenous because multiple antagonistic reactions between different populations occur (Bidle and Azam, 2001; Long and Azam, 2001; Grossart et al., 2004). Although particles have been suggested to serve as microbial refuges from phages (Grossart and Tang, 2010; Tang et al., 2011), they have also been shown to harbor large numbers of viral particles and activities (Proctor and Fuhrman, 1992; Peduzzi and Weinbauer, 1993; Müller-Niklas and Schuster, 1994; Riemann and Grossart, 2008). Until now, relatively few studies have followed temporal changes in bacterial community composition on particles (Grossart and Simon, 1998a; Schweitzer et al., 2001; Grossart et al., 2003a; Kiørboe et al., 2003; LeCleir et al., 2014; Datta et al., 2016), and even fewer studies tried to link it to activity on particles (Schweitzer et al., 2001; Kong et al., 2013; Moran et al., 2013; Smith et al., 2013; D’Ambrosio et al., 2014). Most of these studies focus on single-point measurements and thus do not provide any temporal resolution. Those that do, however, have used closed rolling tank systems (Grossart and Simon, 1998a; Schweitzer et al., 2001; Datta et al., 2016), and are likely to be affected by a series of phenomena summed under the term “the bottle effect” (Zobell and Anderson, 1936). These include a rapid shift in microbial community composition and an increase in heterotrophy vs. autotrophy (Ferguson et al., 1984; Calvo-Díaz et al., 2011; Baltar et al., 2012).

**FIGURE 1:**
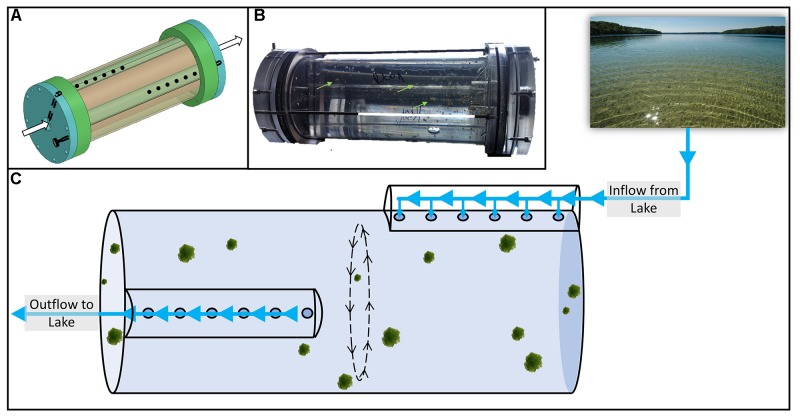
Schematics of the flow-through rolling tank system **(A)** with live diatom particles during an experiment **(B)**. The conceptual experimental setup is shown in panel **(C)**. The incubation chamber of the flow-through rolling tank is 9 cm in diameter, 30 cm long and has a volume of ca. 1.9 L. Extensive technical details are given in Ionescu et al. (2015).

**FIGURE 2:**
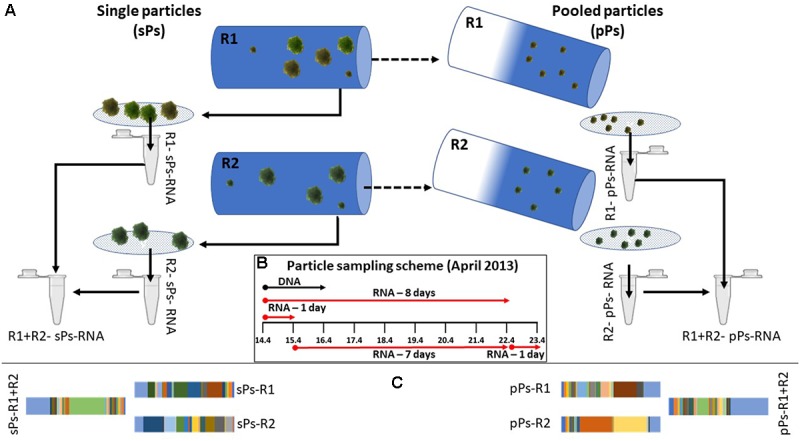
The sampling procedure **(A)** included collection of 3–4 particles (referred to, when pooled, as single particles; sPs) from parallel incubating rolling tanks (marked here as R1 and R2 for replicates 1 and 2) after which the remaining water, pooling lake particles (referred to as pooled particles; pPs) and microscopic fragments of inoculum particles were filtered on a 5 μm pore size filter. RNA was extracted and mRNA was enriched from each fraction collected. Pooled samples consist of equi-volume mixing of mRNA of two single particle or two pooled lake particles samples. The sampling schemes **(B)** depict the start and end of incubation times in April 2013. Samples were collected at the end of the incubation period. A subset of the functional annotation results is shown, demonstrating the different results obtain from single sample vs. pooled sample analysis in both single particles and pooled lake particles **(C)**. In subsequent figures the first and second starting replicates of each incubation type (long or short) are named R1–R4 for replicates 1–4.

To overcome this “bottle effect” and allow for constant exchange of nutrients and microorganisms from the environment, we connected flow-through rolling tanks (Ionescu et al., 2015; Figure 1) directly to the meso-oligotrophic Lake Stechlin (northeastern Germany). The tanks were inoculated with pre-formed particles (see section “Materials and Methods”) of an axenic diatom (*Navicula* sp.) previously isolated from the lake. Particles were collected at several defined time points for respiration and photosynthesis measurements (Ionescu et al., 2015), bacterial community composition, metagenomic, and metatranscriptomic analyses of replicates of 3–4 pooled particles (hereafter single particles). These were further compared to hundreds to thousands of pooled particles (hereafter pooled particles; Figure 2) by filtering the ambient water in the flow-through reactor. Last the single-particles and pooled-particle samples were compared to the results obtained when pooling RNA from two biological replicates. First results (Ionescu et al., 2015) show that under near *in situ* conditions, and as long as light is available, photosynthesis remains active for up to 9 days, twice as much as in a closed rolling tank. Accordingly, given continuous production of photosynthesis products during these 9 days as well as an expected continuous supply of fresh labile organic matter from the lake, respiration on particles remains high under near *in situ* conditions for the whole duration of the experiment. This is in clear contrast to a closed incubation system, which excludes the supply of fresh, labile organic matter and thus reveals a rapid decrease in respiration with time (Ionescu et al., 2015).

We hypothesize that: (i) Community composition as well as transcriptomic profile of pooled particles differ from those of individual particles; (ii) In the photic zone, changes in carbon-quality, as can be interpreted by transcriptomic profile of photosynthetic particles are slow due to active photosynthesis and constant supply of fresh, labile organic matter thus minimizing carbon-quality-based changes in the particle-associated microbial community composition; (iii) Initial microbial colonization is random and; (iv) early changes in microbial communities on particles are mainly due to dynamics in microbial interactions.

Our data calls for modifications of current sampling and processing strategies of particles. Individual particle studies are required to address the degree of heterogeneity in microbial dynamics on particles of similar (e.g., homogenous blooms) or diverse organic matter sources.

## Materials and Methods

### Algal Culture

An axenic culture of the freshwater diatom *Navicula* sp., isolated from Lake Stechlin, was grown in 900 mL of sterile *Z*-medium (Staub, 1961) in 10 sterile, pre-combusted 1 L Duran bottles (Schott). The culture was grown under a 12 h dark-light cycle at 15°C and 30 μmol photons m^-2^ s^-1^ for 2 weeks. Afterward, bottles with axenic diatoms were placed on a roller table at half the light intensity for particle formation prior to *in situ* inoculation with freshwater bacteria from Lake Stechlin.

### Experimental Setup

To conduct long-term experiments with a constant supply of fresh OM, nutrients, and bacteria from the habitat while avoiding the “bottle effect” we used a newly designed flow-through rolling tank system (Ionescu et al., 2015; Figure 1A). Shortly, this device consists of an inner tube in which the continuous sinking of particles is simulated and an external casing through which lake water flows in and out of the inner tube without disturbing the particles (Figure 1B). A detailed description necessary for construction of the flow-through rolling tank is available in Ionescu et al. (2015).

Freshly formed axenic diatom particles were cautiously transferred to the prefilled inner core of the rolling tank device carefully avoiding air bubbles. The axenic nature of the diatom culture renders fresh particles rather fragile (Gärdes et al., 2011). Thus, since some particles may break apart and exit the system, the flow-through rolling tank was inoculated with ca. 100 particles 1–2 mm in diameter each. The device was then sealed and allowed to roll for up to an hour prior to onset of lake water flow. Lake water was pre-filtered through a 100 μm pore size sieve to prevent large objects including mesozooplankton from entering the experimental setup. Flow was maintained at ca. 300 ml h^-1^ and rotation speed was set at 2 RPM keeping the particles suspended and without contact with the walls of the flow-through rolling tank, mimicking a sinking speed of ca. 7 m d^-1^.

### Sampling Procedure (Figure 2A)

Each rolling tank was opened, and 3–4 individual *Navicula* particles (referred to as single particles when pooled) were carefully sampled using a cut-end syringe and avoiding collection of surrounding water. Collected particles were rapidly gathered and placed on a 5 μm pore size polycarbonate filter (47 mm in diameter, Sartorius, Germany) to remove excess water and free-living bacteria. Subsequently, the filters were immediately placed in Z6-buffer (8 M guanidinium-HCl, 20 mM MES, 20 mM EDTA [pH 7.0], and 0.7% [v/v] 2-mercaptoethanol) to deactivate RNAses and denature all proteins. To collect the lake particles that include particle from the lake and microscopic particles from the *Navicula* innoculum, the total water volume of each rolling tank (1.8 L) was filtered through 5 μm pore size filters. Filters were changed each time the filtration speed slowed down to avoid clogging and collection of free bacteria on the pooled lake particles (**pooled lake particles**). All filters were immediately placed in 700 μL of Z6 buffer and placed on ice for further analyses.

A total of eight individual rolling tanks were set up for the experiment forming four biological replicate experiments for short and long incubation each as depicted in Figure 2B. Initially, four reactors were run, two of which were sampled after 24 h and two after 8 days. Two additional reactors were inoculated and run for 7 days once the first set had been sampled. A second two-reactor set of one-day incubations was inoculated on the 8th day of the experiment thus testing for specific colonization of particles, given an 8-day time gap.

### DNA and RNA Extraction

Nucleic acids were extracted as described for DNA in Ionescu et al. (2012), except for the last desalting step. Shortly, cells were lysed by incubation for 15 min. at 95°C in a Z6-buffer followed by 15 min. incubation at 65°C with phenol (0.5:1 phenol:sample, v:v; Phenol pH 8 and 4.3 for DNA and RNA, respectively) after which the aqueous phase was extracted with chloroform. After an additional phenol-chloroform (1:0.5:0.5, sample:phenol:chloroform, v:v:v) extraction, the samples were extracted twice with chloroform and precipitated over night at -20°C using 2-propanol (1:1). The DNA was cleaned in 70% ethanol and dissolved in Diethylpyrocarbonate (DEPC) treated water. Following the extraction, RNA, samples were treated with Turbo DNA Free (Ambion) according to the manufacturer’s instructions.

### mRNA Enrichment and cDNA Synthesis

Since ribosomal RNA is the most common transcript in RNA extractions we have enriched the mRNA by removing all rRNA transcripts following the procedure described in Stewart et al. (2010). Shortly, we generated long 16S and 23S rRNA probes by obtaining sample specific rRNA gene PCR amplicons which were later transcribed to biotin labeled sample-specific RNA probes using the MegaScript T7 kit (Ambion) and biotin labeled C and U (Roche). To achieve maximum rRNA removal, all probes from all samples were pooled and equal aliquots were added to the RNA samples. Following an incubation of 5 min. at 70°C, the hybridized rRNA molecules were extracted with streptavidin coated magnetic beads. The mRNA enriched RNA was concentrated by using the Easy RNA kit (Qiagen). First strand cDNA was synthesized using the Superscript III kit (Invitrogen) according to the manufacturer’s instructions.

Pooled samples consist of an equal volume of mRNA-enriched RNA from a pair of individual samples of either single particles or pooled lake particles. Equal volumes were chosen over equal RNA concentrations to better represent particles of similar sizes colonized at different microbial densities with additionally different level of activity.

### Sequencing

#### Shotgun Sequencing From DNA

Metagenome sequencing steps included fragmentation of genomic DNA, ligation to sequencing adapters and purification. Following the amplification and denaturation steps, libraries were pooled and sequenced. We used 50 ng of DNA from each sample to prepare the libraries using Nextera DNA Sample Preparation Kit (Illumina). Library insert size was determined by Experion Automated Electrophoresis Station (Bio-Rad). The insert size of the libraries ranged from 300 to 1400-bp. The pooled library (12 pM) was loaded to a 600 Cycles v3 Reagent cartridge (Illumina) and the sequencing was performed on a Miseq (Illumina) sequencer. Sequencing was carried out at Molecular Research Laboratories (Mr. DNA), Shallowater, Texas.

#### cDNA Sequencing

cDNA sequencing steps include purification and generation of blunt end cDNA followed by ligation to sequencing adapters, amplification, denaturation, and sequencing. We used 250 ng of double strand cDNA from each sample to prepare the libraries using TruSeq RNA sample preparation kits (Illumina). The pooled library (10 pM) was loaded to a 600 Cycles v3 Reagent cartridge (Illumina) and the sequencing was performed on Miseq (illumina). cDNA sequencing was carried out twice, the first-time biological duplicates were pooled and the second time each sample was sequenced individually. The results of the first sequencing are provided as Supplementary Information. Sequencing was carried out at Molecular Research Laboratories (Mr. DNA), Shallowater, Texas.

#### Sequence Quality Control

Sequence quality was assessed using the FastQC program (Babraham Institute) after which each sequence file was cleaned from low quality sequences and traces of the sequencing method with the Clip tool from the Nesoni package (Victoria bioinformatics) using the following parameters: the first 12 and last 3 bases were trimmed and sequences shorter than 50 nucleotides were removed; from the remaining data, sequences with an overall quality score below 20 were removed as well.

### Sequence Analysis

The metagenome was analyzed on the MG-RAST server (Meyer et al., 2008).

#### Transcriptome

All paired end and single end libraries from the transcriptome analysis generated during the sequence quality control procedure were concatenated into three study-wide data files: left, right and non-paired reads, to maximize the chances of transcript assembly.

Transcripts were assembled and analyzed using the Trinotate pipeline^1^. This includes transcript assembly with the Trinity pipeline (Grabherr et al., 2011b), open reading frame prediction using TransDecoder (part of the Trinity software package), protein identification using BlastX and BlastP (Altschul et al., 1990) against the Uniprot protein database (The uniProt Consortium, Bateman et al., 2017), protein domain prediction using HMMER (V3.1; Finn et al., 2011) against the Pfam database (Finn et al., 2014). To obtain additional annotation data, all transcripts were run through the KEGG KAAS system (Moriya et al., 2007). The basic analysis pipeline is amended with data analysis against functional databases (CAZY; Lombard et al., 2014), and structural databases (SCOP; Fox et al., 2015 and SUPERFAMILY; Wilson et al., 2009) to maximize annotation of unknown proteins. A prediction of polyketide synthetases and non-ribosomal peptides synthetases was done using a locally installed version of ANTISMASH (Weber et al., 2015) and NaPDoS (Ziemert et al., 2012).

Differential expression analysis was conducted using edgeR (Robinson et al., 2010) (v 3.14) using the FPKM normalization mode (Wagner et al., 2012). The 16 samples (eight in each group: single particles or pooled lake particles) were compared grouped by samples (generating duplicates per condition) or by duration of incubation (short vs. long; generating four replicates per condition). Benjamini-Hochberg false discovery rate test and Bonferoni corrected *p*-values were used to test for statistically significant difference in transcriptomic profile.

The presence of CRISPRs (Clustered Regularly Interspaced Short Palindromic Repeats) was tested using CRISPRfinder (Grissa et al., 2007). Spacer-sequences from candidate CRISPRs were than compared to the entire transcriptome data as well as to the assembled transcripts using Bowtie2 and BLAST, respectively.

### Reconstruction of Bacterial Community

Since rRNA was removed from the samples, microbial community composition was reconstructed from the raw reads as annotated on the MG-RAST server (Meyer et al., 2008) against the M5NR database.

### Species Co-occurrence Networks

Species–species association networks were constructed using CoNet (Faust and Raes, 2016) as implemented in Cytoscape 3.3.0 (Shannon et al., 2003) software. This program was chosen as it allows the merging of networks calculated via several association methods of which we chose: Kendall, Pearson and Spearman correlations, as well as Steinhaus and variance log ratio similarities. The latter results in values between 0 and 1, whereas the rest range between -1 and 1. Only edges confirmed by at least two methods each with a score either smaller than -0.6 (negative association) or larger than 0.6 (positive association) were retained in the final network. The resulting network was visualized and graphically organized using Cytoscape 3.3.0 (Shannon et al., 2003).

### Statistical Analysis

Principle component analysis was done on gene and transcript abundance matrices produced by the Trinotate^1^ pipeline as well as on the function abundance matrix generated by the MG-RAST (Wilke et al., 2016) pipeline. The analysis was done using Primer software version 6 supplemented with the Permanova package. Permanova analysis was conducted on the same data using duration of incubation (short for 1 day or long for 7–8 days) and sample type (single particles or pooled particles). A similar division was used to conduct analysis of similarity (ANOSIM), forming four groups with four member-replicates each. Prior to all analyses the data was square-root transformed and standardized by total reads.

## Results

### Community Composition

#### The Active Bacterial Community (mRNA) on Single Particles Was Heterogeneous and Differed Between Biological Replicates and Incubation Time (Figure 3)

**FIGURE 3:**
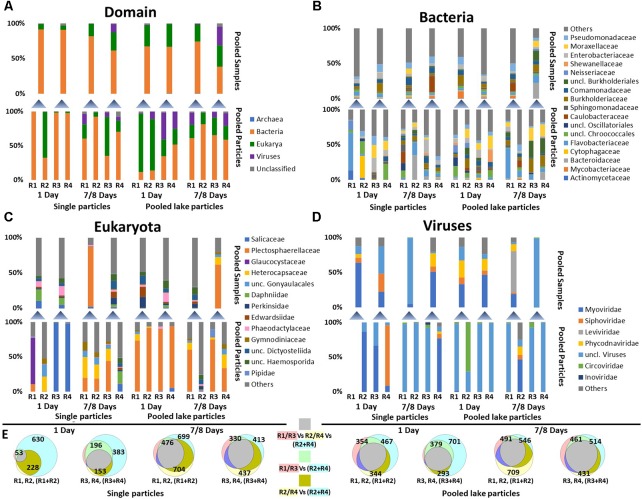
Community composition of single particles (3–4 individual particles pooled) and pooled lake particles according to MG-RAST metagenomics annotation. **(A)** The distribution of Archaea, Bacteria, Eukarya, and Viruses; **(B)** Bacterial families with a sequence frequency of more than 5% in at least one sample; **(C)** Eukaryotic families with a sequence frequency of more than 5% in at least one sample; **(D)** Virus families with a sequence frequency of more than 5% in at least one sample. The lower stacked bars in each panel represent individual samples (Replicate reactors R1–R4) while the upper ones the sequencing results of poled RNA from the pair of sample below. **(E)** Venn diagrams showing the taxonomic overlap between single particle samples (R1–R4) and pooled RNA extracts (R1 + R2 or R3 + R4) at the family level including Bacteria, Archaea, Eukaryotes, and Viruses. The diagrams do not account for abundance but only presence absence. The number of taxonomic entities in each group is shown in each circle of the diagram. The shading color of the text in the legend of the Venn diagram depicts the color of the individual sample circles. The color of squares represents the overlapping between the different samples.

Community data was deduced from the taxonomic annotation of coding RNA (i.e., protein coding), therefore, it is unlikely that it was affected by our rRNA removal protocol. Short-term incubations (1 day), revealed that reads from single particles (3–4 pooled individual particle; see Figure 2 and section “Materials and Methods”) consisted almost exclusively of bacterial sequences almost randomly distributed between the samples. After long-term incubations (7 and 8 days) sequence frequencies of eukaryotes (mostly fungi) and viruses increased (Figure 3A). Whereas sequence frequencies of *Bacteria* on pooled particles (Figure 2) were initially low, they increased in the long-term incubations. Average sequence frequencies of viruses on pooled lake particles remained similar over time (Figure 3A).

#### Microbial Composition of Single Particles Differed From That of Pooled Duplicate Samples Across All Domains (Figures 3A–D)

Initially, identified taxa (families) from single particles represented a small subset of those identified in the pooled samples. In the longer incubations, however, the overlap in present taxa (i.e., presence absence similarity) increased (Figure 3E), but sequence frequencies still played a major role for the observed differences between single and pooled samples.

#### Total Bacterial Communities (DNA) of Both Single Particles and Pooled Lake Particles Were Similar, but Greatly Differed From Active Communities (RNA) (Supplementary Figure S1)

While the total bacterial community on single particles and pooled lake particles was dominated by *Caulobacteraceae*, *Commamonadaceae*, and *Pseudomonadaceae*, these families were either absent or significantly less dominant in the respective active communities (Figure 3B). The dominant active families consisted of *Flavobacteriaceae*, *Pseudomonadaceae*, *Cytophagaceae*, *Enterobacteraceae*, *Moraxellaceae*, *Sphingomonadaceae*, and *Bacteroidaceae* (Figure 2B). Bacterial communities on pooled lake particles, particularly of long-term incubations, were more similar to each other than single particles (Figure 3B). By using rRNA gene amplicon (i.e., DNA) sequencing, Ionescu et al. (2015) showed that the total microbial community in flow-through rolling tanks was rather stable for a long time (>1 week).

The active eukaryotic community from the long-term incubations of single particles and all pooled lake particles was **dominated by *Plectosphaerallaceae*, a family of fungi** (previously identified and isolated from Lake Stechlin; Wurzbacher et al., 2016), and several dinoflagellate families. The eukaryotic community, except for single particles in short-term incubations, was more homogenous than for bacteria (Figure 2C). Pooled lake particles had higher sequence frequencies of eukaryotes (Figure 2A) indicating more diverse source particles. Similar to the active bacterial communities, eukaryotes are highly alike between the total single particles and pooled lake particles communities (DNA), but greatly differed from the active community (RNA).

**Viruses** made up only a small portion of the reads from the short-term incubated single particles and consisted of *Myoviridae*, *Siphoviridae*, and unclassified viruses (Figure 2D). However, with the increase in viral sequence frequency in the long-term incubations, these families were replaced almost entirely by sequences of unclassifiable viral origin and a few *Circoviridae*. Viruses on pooled lake particles remained mostly unclassified, except for one long term incubation revealing mostly *Myoviridae*, *Siphoviridae*, and *Phycodnaviridae*. Interestingly, these virus families were not abundant on the single particles from the same incubation.

### Functional Annotation

The Trinotate assembly pipeline^1^, as applied to the pooled sequences of all samples, (Supplementary Data Sheets 1–3) resulted in 48,787 transcripts larger than 90 nt (30 amino acids), located on 25,049 contigs. Of these, 22,585 transcripts could not be assigned to any potential function by any of the analysis tools used (Supplementary Tables S1, S2).

**FIGURE 4:**
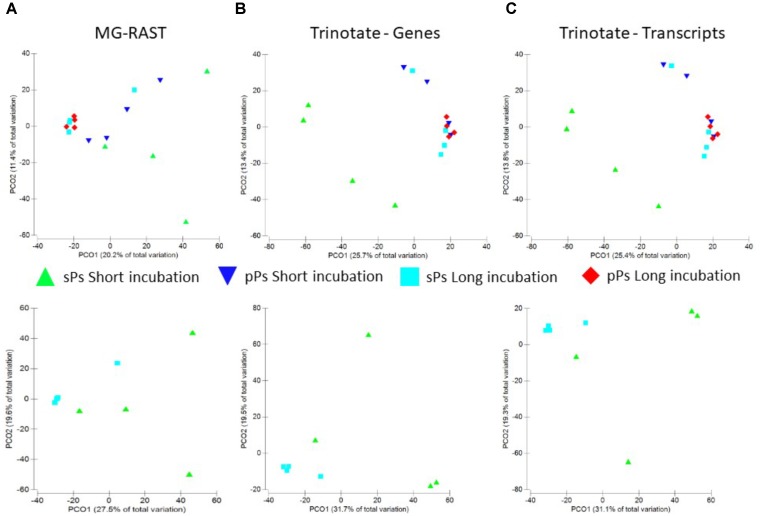
Principle component analysis of raw reads annotation using the MG-RAST pipeline **(A)**, assembled genes **(B)**, and assembled transcripts **(C)** from single and pooled particles (upper panels) or single particles alone (lower panel) grouped by the length of the incubation period where “short” refers to 1 day and “long” to 7/8 days incubations.

One of our major aims was to test whether a rapid change in particle carbon quality also occurs under near *in situ* conditions. This should result in noticeable changes in the particle transcriptome profile and possible in shifts in microbial communities from generalists to more specialized communities. The particle aging period employed here (7–8 days) is on par with other studies reporting changes in expressed gene profiles (Datta et al., 2016; 6 days). Our results suggest grouping of multiple particles masks heterogeneity among particles (Figure 3). Thus, all transcript combinations were analyzed for differential functional gene expression (four short-term single particles vs. four long-term single particles incubations and two short-term pooled particles vs. two long-term pooled particles incubations). These analyses were conducted on annotated transcripts, translated proteins as well as on the raw reads as annotated by MG-RAST (Wilke et al., 2016). In all cases, the graphical representation suggests that the genes expressed on the single particles in the long-term incubations bare more similarity to the long-term incubation in the lake. This makes sense since with time, given the nature of the open system, there would be lake particles adhering to the experimental inoculum as well as the likely establishment of an equilibrium with the lake community. Nevertheless no significant difference in the expression of any gene between the short- and long-term incubations as well as between single and pooled particles occurred when using Bonferroni corrected *p*-values (*p* = 1), Benjamini–Hochberg false discovery rate test (FDR = 0.9–1), or Permanova analysis (Figure 4).

**FIGURE 5:**
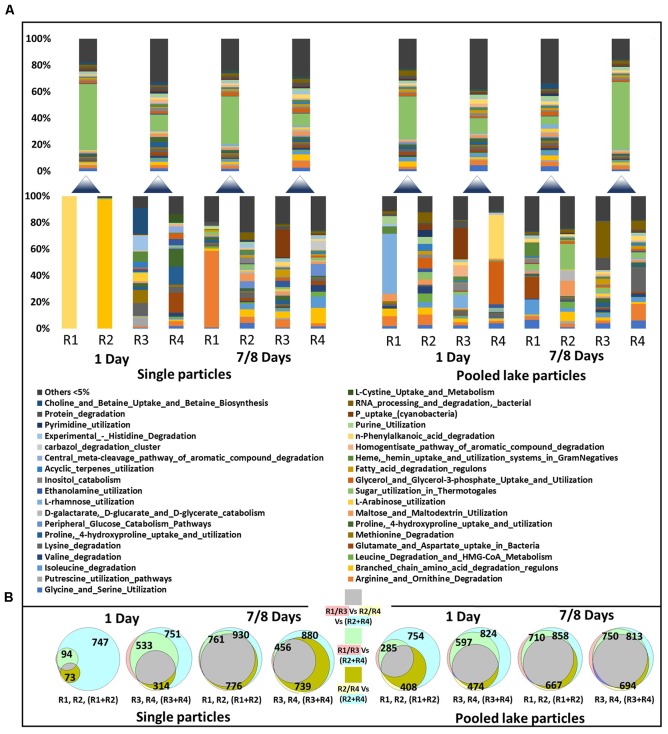
A subset of functional modules involved in substrate degradation and utilization obtained from single particles (3–4 individual particles pooled) and pooled lake particles transcriptomes **(A)** The lower stacked bars represent individual samples while the upper ones the sequencing results of poled RNA from the pair of sample below. The shown modules are Level-3 MG-RAST subsystem hierarchy. Venn diagrams **(B)** show the overlap between all the recognized modules including the subset in panel A, between single particle samples (R1–R4) and pooled RNA extracts (R1 + R2 or R3 + R4). The number of modules identified in the transcriptome of each sample is given in each circle of the diagram. The shading color of the text in the legend of the Venn diagram depicts the color of the individual sample circles. The color of squares represents the overlapping between the different samples.

To test whether changes in expression occur only in the subset of genes specific to enzymatic degradation and substrate utilization these were extracted from the MG-RAST annotation (Figure 5B). A similar approach was applied to sugar, nitrogen, phosphorus, and iron metabolism (Supplementary Figures S2–S5). These analyses showed a large heterogeneity between single particles with no significant difference between young (1 day) or older (7–8 days) particles (ANOSIM *p* => 0.2 in all cases).

Initially, as for the colonizing communities, the functional modules identified on single particles represented a small subset of the pooled single and lake particles (Figure 5A). At the end of the experiment, however, there was a much higher overlap between single particles and pooled single and lake particles.

Considering the ongoing photosynthetic activity of diatoms forming the particles (Ionescu et al., 2015) and the continuous supply of fresh, labile organic matter (both dissolved and particulate) pumped through the vessel from the lake, there are two main explanations for the absence in differences between expression profiles of particle associated communities on fresh vs. aged particles (Figure 5B). First, communities of aged particles still consist of initial colonizers. Second and more likely, competition for resources and other inter-organismic interactions led to a community replacement by others with similar functions. Although our dataset is limited to testing the first possibility as individual particles couldn’t be sampled at multiple time points and particle numbers were insufficient for a proper statistic, we searched for indications of inter-organismic interactions.

### Possible Evidence for Antagonistic Reactions

#### Anti-phage Activity

**Clustered Regularly Interspaced Short Palindromic Repeats (CRISPR)** sequences consist of several similar stem-and-loop palindromic sequences separated by spacers of different sequence (Sorek et al., 2008). To identify potential CRISPR sequences, we analyzed the metagenomes of single particles and pooled lake particles and revealed 735 putative CRISPRs including 1066 different spacers (Supplementary Table S3; Supplementary Data Sheet 4). Among the metagenomic CRSIPRs 222 had also matches in the assembled transcriptome. Additionally, we found the expression of different CRISPR associated genes including Cas1, Cas2, Cas3, and Cas6.

A second defense mechanism employed by bacteria against foreign viral DNA is the use of **restriction endonucleases (restriction enzymes;** Weinbauer, 2004). Overall multiple Type I, II, IIS, and III endonucleases were identified in the Trinity (Grabherr et al., 2011a) assembled transcripts and the MG-RAST analysis.

#### Interactions Between Other Organisms

In addition to viruses, further interactions between organisms such as antagonistic interactions between bacteria or prokaryotic and eukaryotic organisms affect microbial community composition (Grossart et al., 2004). Such interactions are evident in the expression of protein secretion system type II, III, IV (Supplementary Data Sheet 1), and VII (Supplementary Data Sheet 2), genes involved in antibiotic production and defense mechanisms as well as the expression of toxin-antitoxin systems (Supplementary Data Sheets 1, 2). Several variants of polyketide synthases and non-ribosomal peptide synthetases were found as well (Supplementary Table S4).

## Discussion

Our study using an open, flow-through particle incubation system that significantly minimizes or removes the “bottle effect” portrays the important role of particles as surfaces and hotspots for microbial colonization, metabolic activity, and interactions in the water column. First, we show a large heterogeneity between microbial communities attached to single particles of identical source and age. Second, we show a high heterogeneity in specific activities on these particles. Last, expression of genes known to be involved in antagonistic reactions between organisms including viral and anti-viral activity highlight the importance of organismic interactions for microbial community dynamics on particles. These findings highlight particles as hotspots of biogeochemical organic matter cycling in the water column with profound consequences for the microbial carbon pump (Jiao and Azam, 2011) and overall carbon pump efficiency (Legendre et al., 2015). Furthermore, differences between single particles, pooled lake particles and pooled samples highlight pitfalls to mechanistically understand temporal dynamics of microbial particle degradation when averaging samples. Averaging multiple particles of different ages and sources as is the case in nature can only provide in inventory of organisms and processes occurring on particles. As we show, each individual particle contributes only a subset of this information providing specific information on the microbial assemblages and their particle-degrading activity.

Particle type as a proxy for carbon quality has been suggested to play a significant role in composition and activity of the attached bacterial community (Grossart et al., 2005; Teeling et al., 2012). Datta et al. (2016) by incubating particles in a closed system demonstrated that microbial colonization and succession of total communities (DNA based) on artificial particles of the same type are reproducible. Our study, minimizing the “bottle-effect,” demonstrates that, under near *in situ* conditions, particle associated communities on single particles of identical source have a high inter-particle heterogeneity. This heterogeneity between biological replicates indicates that the nature of the initial particle colonizing community is not a sole function of particle type and composition, but also of other – perhaps partially stochastic events (Cordero and Datta, 2016; Datta et al., 2016), as discussed below. This suggests that in any given environment some members of the residing microbial community will be able to colonize particles. When extrapolating to the natural aquatic environment, where particles of multiple origins are simultaneously formed, it is likely that these will be rapidly and differently colonized regardless of the surrounding microbial community.

Under near *in situ* conditions, the initial degradation process does not seem to select for specific microbial groups. On aged particles (7–8 days), microbial communities are as heterogeneous as on “fresh” particles (1 day). The higher similarity between pooled lake particles suggests that heterogeneity between individual particles is masked by averaging over large volumes (i.e., ∼2 L in this case) encompassing hundreds of particles of variable type and age. A previous study comparing, using microscopy, microbial colonization on several thousands of individual particles collected from a single environment – similarly reported a large heterogeneity between particles (Bižić-Ionescu et al., 2015).

Conventional molecular studies on particles have mainly used size differential filtration to obtain various particle size classes (Padilla et al., 2015). Thus, particles are usually treated as an integral of various entities with unknown history and chemical quality ignoring almost completely inter-particle heterogeneity. Our study demonstrates for both microbial community composition and activity, as reflected by transcriptomic profiles, that the sum of two samples, either of single particles or of larger volumes (pooled samples of already pooled lake particles) differs from single particles, in particular during early colonization. This is probably the result of inter-particle heterogeneity in microbial community composition, activity, and colonization density. Furthermore, numerous pooled particles mask the composition and activity of specific subsets of the community associated with individual particles. Therefore, sample or particle pooling does not allow for an understanding of microbial interactions involved in the degradation of a particle of a specific age and/ or source. As particles age, they seem to be more homogenous both in community composition and functionality. Therefore, samples consisting of fresh and aged particles are even more problematic for understanding the initial steps of particle colonization and degradation. To better understand bacterial colonization and succession on particles in relation to their specific organic matter quality, either the use of model particles and organisms is required (Enke et al., 2018) or metagenomic and metatranscriptomic studies of individual single particles with well-defined organic matter composition are required. Findings from individual particles of similar age and composition are likely to reveal functional redundancy among individual microorganisms as well as between different assemblages. Additionally, information on microbial assemblages from individual particles will show if certain microorganisms are exclusively mutualistic and perhaps actively recruit one another from the water column, while others always exclude one another.

### Potential Determinants of Particle-Associated Microbial Community Composition

#### High Colonizer Patchiness

Stochasticity of attachment to surfaces, based on the “first comes, first served” concept may play a major role in determining the composition of the initial particle associated bacterial community. Bacterial distribution in water is generally patchy (Cordero and Datta, 2016). The presence of particles may further affect this patchiness by releasing dissolved organic matter and nutrients and thus attracting different chemotactic bacteria (Kiørboe et al., 2002, 2004; Stocker, 2012). Bacterial populations differ in their affinity to the substrates released from particles, and consequently different particles may attract and be colonized subsequently by different bacteria.

#### Colonizer–Particle Interactions Differ Between Individual Particles

Microbial utilization of particle resources is unlikely to occur without any competitive interactions between the numerous potential groups of colonizers (Grossart et al., 2003a,b). Studies have shown *in vitro* antagonistic behavior between isolates obtained from particles (Long and Azam, 2001) and competitive particle colonization (Grossart et al., 2003b). Interactions can occur bi-directionally between different populations: (a) populations of eukaryotes such as particle-forming algae and colonizing fungi or among populations of colonizing fungi; (b) bacteria and eukaryotes such as particle-forming algae and colonizing bacteria (Amin et al., 2012) or between colonizing bacteria and fungi; (c) multiple colonizing bacterial populations including protozoan grazers (Long and Azam, 2001; Grossart et al., 2003b), and (d) colonizing organisms and viruses (Riemann and Grossart, 2008). However, solely analyses of single particles have the potential to identify antagonistic interactions between various colonizing organisms and thus can discriminate the role of particle age and quality from other factors. Accordingly, our data support the presence of competitive microbial interactions, e.g., the attempt of particle-associated bacteria to inhibit the particle-forming diatom.

Bacterial – host antagonism is indicated by the expression of type II, III, IV, and VII protein secretion systems (Supplementary Data Sheets 1, 2). Furthermore, evidence for toxin production in our transcriptome alongside with various resistance mechanisms (Supplementary Data Sheets 1, 2) indicate a plethora of antagonistic interactions. For example, genes involved in the synthesis of phenazine, a toxic *Pseudomonas* pigment (Gibson et al., 2009), were also found among the particle transcripts. This pigment, both in natural and modified forms, was shown to be toxic for several eukaryotic organisms (Cezairliyan et al., 2013).

#### Multiple Bacteria–Bacteria Interactions

We detected the expression of a few genes directly involved in the synthesis of known antibiotic compounds such as mitomycins and carbapenem as well as several genes predicted to be involved in antibiotic production. A search for polyketide synthetases and non-ribosomal peptide synthetases showed a few matches as well (Supplementary Table S4). On the other hand, numerous genes such as efflux pumps, transporters, and hydrolases associated with resistance to antibiotics such as polymixin, erythromycin, spectinomycin, and streptomycin occur on particles. This supports our hypothesis of an antagonistic and hence partly competitive behavior between different particle colonizing microorganisms (e.g., bacteria and fungi).

The calculation of co-occurrence networks from the single particles associated communities (Supplementary Figure S6) showed that there is no overlap in species associations between short- and long-term incubations. Similarly, no overlap is observed between the single particles and the pooled lake particles networks (data not shown). Hence, while each network in its own suggests that some species co-occur while other are mutually absent, these interactions seem to be highly particle specific.

#### Intense Bacteria–Virus Interactions

Bacterial and eukaryotic viruses are strong regulators of microbial communities (Weinbauer, 2004) including those associated to particles. The strong increase in viral sequences on single particles in the long-term incubations indicates that as initial populations settle on the particles they become subject to viral attacks. This is further evidenced by the expression of CRISPRs, the bacterial “immune system.” The constant presence of viruses on the readily colonized pooled lake particles further strengthens the significant, yet underestimated role viruses play on particles. Our microscopic analysis of the incubated particles has detected cells with viral like entities on them (Supplementary Figure S7).

#### No Changes in Microbial Carbon Usage During the Incubation Period

A sequential change in carbon quality is commonly believed to control bacterial successions on particles during a few days of incubation. Nevertheless, indications for such changes come from studies conducted in fully or partially closed systems. In such systems, the bacterial community is strongly altered by the “bottle effect” leaning toward heterotrophy (Sherr et al., 1999; Calvo-Díaz et al., 2011), and naturally occurring DOM is not continuously available. Contrary to these studies, Ionescu et al. (2015) have shown that employing an open (flow-through) experimental system as used in this study, the decrease in algal activity due to bacterial degradation occurs at least 4 days later than in a closed system. Furthermore, respiration on particles incubated in the open system was maintained at a high level for a long period suggesting a labile carbon source was readily available. Although heterogeneity in particle-associated communities was high, we could not detect any (statistically significant) change in the expression of genes, particularly those related to carbon usage. For natural systems, we thus propose that labile carbon is readily available during particle sinking through the upper water layers. Particle sinking velocities range from as low as 1 cm d^-1^ (Estrum-Yousef et al., 2000) to up to at least 250 m d^-1^ (McDonnell and Buesseler, 2010). Hence, some particles will remain in the surface layers of aquatic bodies for rather long periods. However, for a substantial fraction of particles 7–8 days (the duration of our experiment) is too long to stay in the water column of a shallow water body, and surely not in the photic zone of even the open ocean. Studies employing image analysis (Ratmeyer and Wefer, 1996; Nowald et al., 2009), echo sounding systems (Jackson and Checkley, 2011) or sediment traps (McDonnell and Buesseler, 2010) to follow particles throughout the water column, did not find a conclusive decrease in particle size with depth. Thus, while organic matter from photosynthetic activity is no longer available, labile organic matter can reach particles from the dissolved organic matter pool of the surrounding water or from adherence of additional particulate OM.

### Hotspots of Antagonism as a Source of Genetic Variability

The high number of genes involved in inter-organisms interactions and the strong evidence for viral presence and activity support particles being a hotspot for lateral gene transfer (Riemann and Grossart, 2008; Walsh et al., 2013). The phage-mediated transfer of genetic material between different bacteria in aquatic environments is thought to be limited by host abundance in the water column (Weinbauer, 2004). This would probably not be the case on the limited surface of densely colonized particles, where close vicinity and high number of possible vectors may result in numerous transduction events. Our transcriptomes include both Type II and Type IV pili secretion systems needed for the uptake of naked DNA, as well as the recA and recR recombination proteins. Additionally, we found the expression of the *comEA* genes which encode for a protein essential for DNA uptake (Provvedi and Dubnau, 1999). Thus, in addition to gene exchange mediated by virus between (usually) closely related organisms, uptake of naked DNA can also increase genetic exchange between even phylogenetically and functionally distant microorganisms.

## Conclusion

**FIGURE 6:**
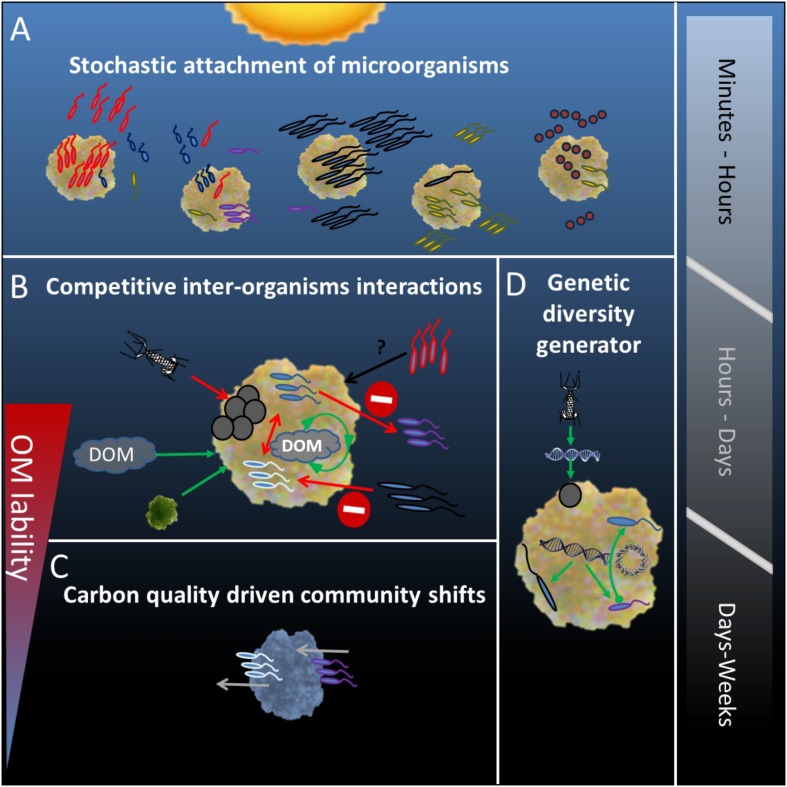
Conceptual summary of events determining the particle associated microbial community composition, from the stochastic attachment of present bacteria in the water column **(A)** through competitive and viral interactions **(B)** to carbon-quality driven changes in community **(C)**. The sequence of events occurring on the particle renders it a generator of genetic diversity through transduction, conjugation, and uptake of naked DNA **(D)**. Microbial attachment is likely to occur rapidly within hours from particle formation. For as long as the particle received internal and external supply of dissolved organic matter, interactions between organisms will determine the community compositions. Depending of the particle’s sinking rate this is a matter of hours to days. Once the supply of fresh organic matter is depleted and the particle’s labile organic matter is consumed carbon-quality driven community shifts will occur, a process that will take place within days from particle formation.

Our study demonstrates that single particles represent complex micro-niches in the water column which are randomly colonized by a diverse microbial assemblage from the surrounding water (Figure 6A). We propose that competitive and antagonistic activities on particles, between all prokaryotic and eukaryotic entities play an essential, yet underestimated role in determining dynamics of particle-associated community composition (Figure 6B). Depending on particle sinking rate, the available labile organic matter can be continuously replenished by internal and external processes. Thus, carbon driven changes in microbial community composition and activity, as derived from experiments using closed incubation devices, are absent or less pronounced in the upper (euphotic) water layers (Figure 6C). It is obvious that different dynamics in organic matter composition of particles incubated in closed or open systems result in different conclusions on microbial community dynamics both in relation to community composition and activity. Thus it is important to better understand and model microbial carbon pump efficiency as current global climate change will result in an increased retention time of particles in the surface layers of water bodies (Legendre et al., 2015). Continuous inter-organism activities on particles render them generators of genetic diversity in aquatic ecosystems (Figure 6D) with increased horizontal gene transfer between particle-associated organisms as compared to their free-living counterparts (Arias-Andres et al., 2018). Studies averaging over multiple particles are valuable in providing a broad catalog of organisms and functions related to particles but are restricted in providing information on specific particle associated consortia and their functions. Therefore, while “All roads may lead to Rome” individual members and physiological processes may be deduced only from focused studies using single particles.

## Author Contributions

MB-I designed the experiments, analyzed the data, and wrote the paper. DI designed the experiments, analyzed the data, and wrote the paper. H-PG wrote the paper.

## Conflict of Interest Statement

The authors declare that the research was conducted in the absence of any commercial or financial relationships that could be construed as a potential conflict of interest.

## Footnotes

1 https://trinotate.github.io/

## Supplementary Material

FIGURE S1 Composition of the total microbial community (DNA) of single particles and pooled lake particles as obtained from a metagenomic analysis of a sample collected after 2 days of incubation.

FIGURE S2 A subset of functional modules involved in sugar utilization obtained from the singe particles and pooled lake particles transcriptomes. The shown modules are Level-3 MG-RAST subsystem hierarchy. Abundance refers to percent of reads out of total attributed to sugar utilization.

FIGURE S3 A subset of functional modules involved in nitrogen metabolism and utilization obtained from the single particles and pooled lake particles transcriptomes. The shown modules are Level-3 MG-RAST subsystem hierarchy. Abundance refers to percent of reads out of total attributed to nitrogen metabolism. Note that subsystem “ZZ gjo need homes” is a valid subsystem name which includes genes with no other subsystem affiliation. In this case, however, the function of genes is associated with nitrogen. The fourth replicate of long incubation single particles (R4) resulted in no transcripts related to nitrogen metabolism.

FIGURE S4 A subset of functional modules involved in phosphorus metabolism and utilization obtained from the single particles and pooled lake particles transcriptomes. The shown modules are Level-3 MG-RAST subsystem hierarchy. Abundance refers to percent of reads out of total attributed to phosphorus metabolism. The fourth replicate of long incubation single particles (R4) resulted in no transcripts related to phosphorus metabolism.

FIGURE S5 A subset of functional modules involved in iron metabolism and utilization obtained from the single particles and pooled lake particles transcriptomes. The shown modules are Level-3 MG-RAST subsystem hierarchy. Abundance refers to percent of reads out of total attributed to iron metabolism. The second replicate of short incubation single particles (R2) resulted in no transcripts related to iron metabolism.

FIGURE S6 Co-occurrence networks of communities associated with single particles at the family level from short- and long-term incubations as calculated using CoNet (Faust and Raes, 2016). Depicted nodes are supported by at least 3 correlation or similarity methods. Node size represents maximal abundance (percent of reads attributed to taxa). Flavobacteriaceae, Moraxellaceae, and Pseudomonadaceae are the only common species between these networks, however, without common associations to other species.

FIGURE S7 Scanning electron micrograph of an individual particle from Day 1 (A) or Day 5 (B). Phage-like particles were observed on cells resembling Caulobacter (C) as well as on other bacteria (D). Red and white arrows represent potential exit sites of phage particles and attached phages, respectively.

TABLE S1 Statistical overview of the transcriptome annotation of all individual samples using the extended Trinotate pipeline.

TABLE S2 Statistical overview of the transcriptome annotation of pooled duplicate samples using the Trinotate pipeline.

TABLE S3 List of transcripts in which CRISPR spacers were identified. Spacers location and coverage details are provided as well. Spacer sequences are given in Supplementary Data Sheet S4.

TABLE S4 Transcripts containing matches to polyketide synthethases and non-ribosomal peptide synthetases.
