# Supplementary figures and images for: Organic Particles: Heterogeneous Hubs for Microbial Interactions in Aquatic Ecosystems
Source: Front Microbiol. 2018 Oct 26;9:2569. doi: 10.3389/fmicb.2018.02569 (PMC6212488; doi:10.3389/fmicb.2018.02569)

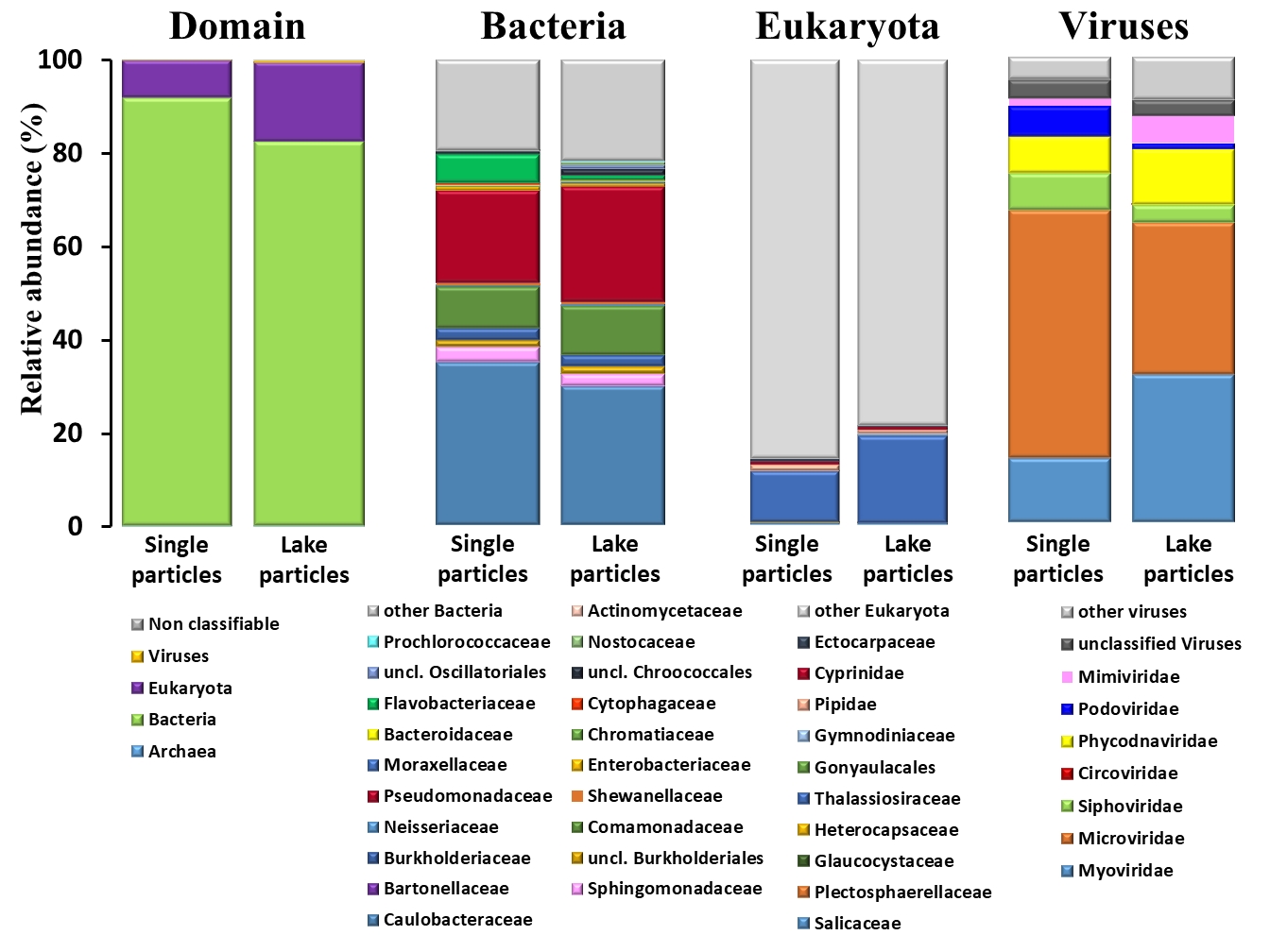

Supplement: FIGURE S1 — Composition of the total microbial community (DNA) of single particles and pooled lake particles as obtained from a metagenomic analysis of a sample collected after 2 days of incubation. [file Image_1.JPEG]

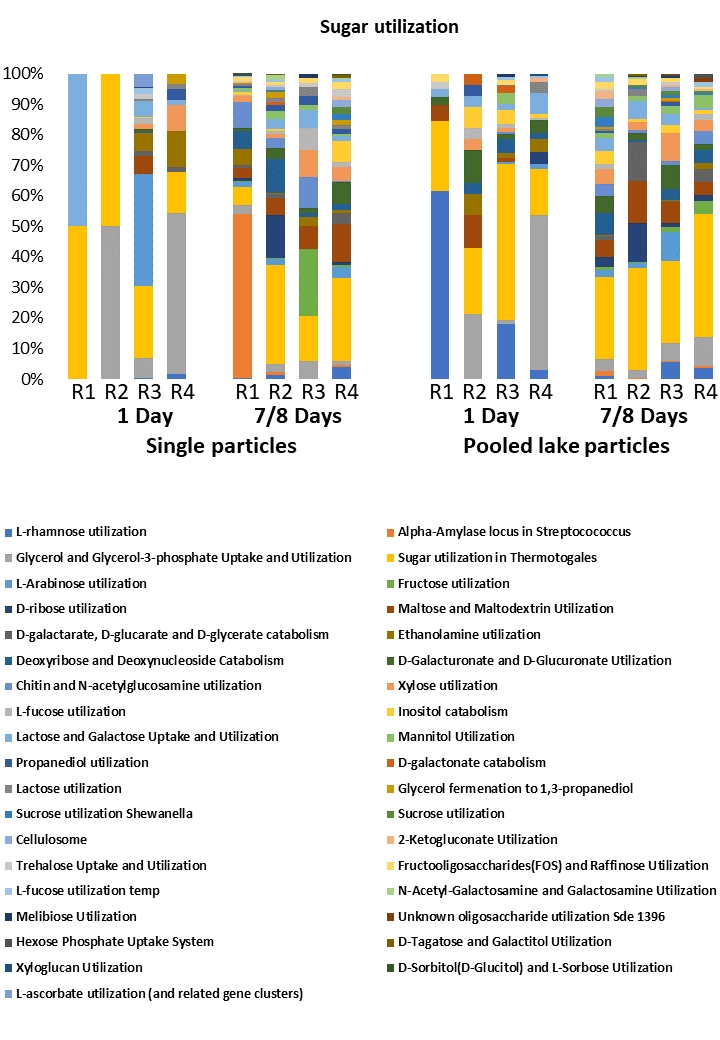

Supplement: FIGURE S2 — A subset of functional modules involved in sugar utilization obtained from the singe particles and pooled lake particles transcriptomes. The shown modules are Level-3 MG-RAST subsystem hierarchy. Abundance refers to percent of reads out of total attributed to sugar utilization. [file Image_2.JPEG]

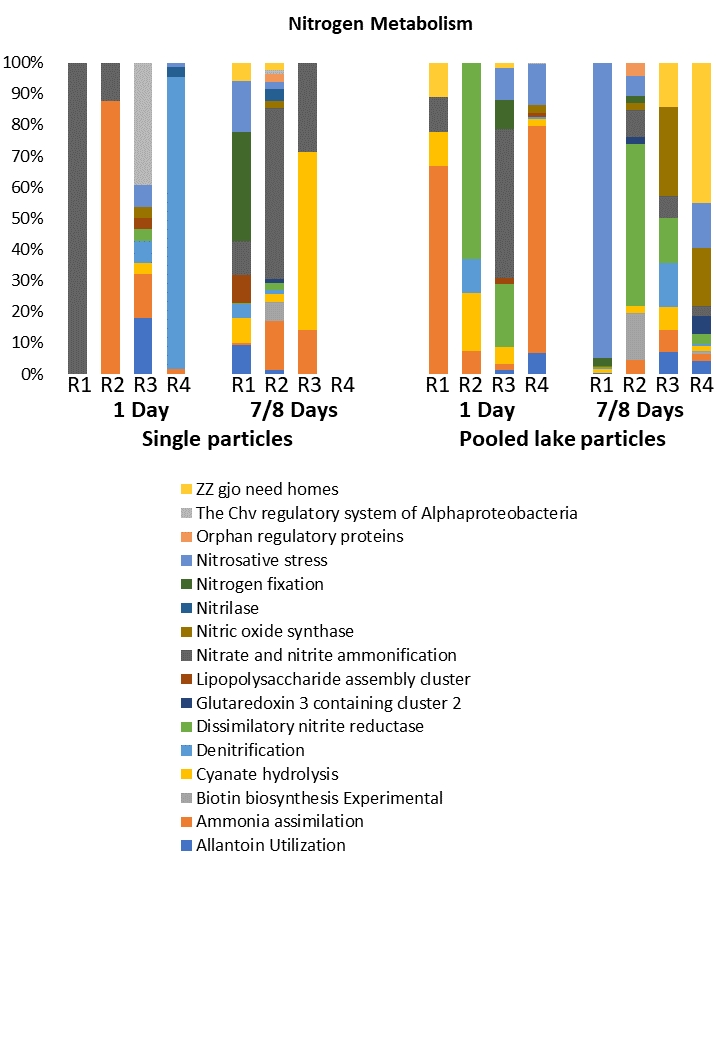

Supplement: FIGURE S3 — A subset of functional modules involved in nitrogen metabolism and utilization obtained from the single particles and pooled lake particles transcriptomes. The shown modules are Level-3 MG-RAST subsystem hierarchy. Abundance refers to percent of reads out of total attributed to nitrogen metabolism. Note that subsystem “ZZ gjo need homes” is a valid subsystem name which includes genes with no other subsystem affiliation. In this case, however, the function of genes is associated with nitrogen. The fourth replicate of long incubation single particles (R4) resulted in no transcripts related to nitrogen metabolism. [file Image_3.JPEG]

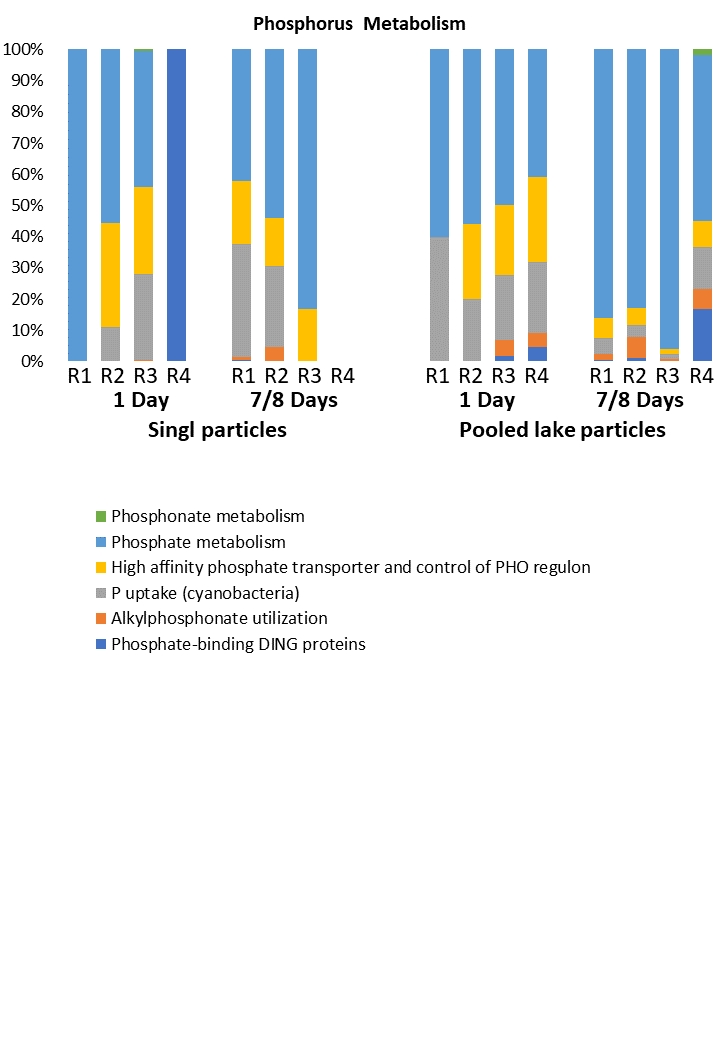

Supplement: FIGURE S4 — A subset of functional modules involved in phosphorus metabolism and utilization obtained from the single particles and pooled lake particles transcriptomes. The shown modules are Level-3 MG-RAST subsystem hierarchy. Abundance refers to percent of reads out of total attributed to phosphorus metabolism. The fourth replicate of long incubation single particles (R4) resulted in no transcripts related to phosphorus metabolism. [file Image_4.JPEG]

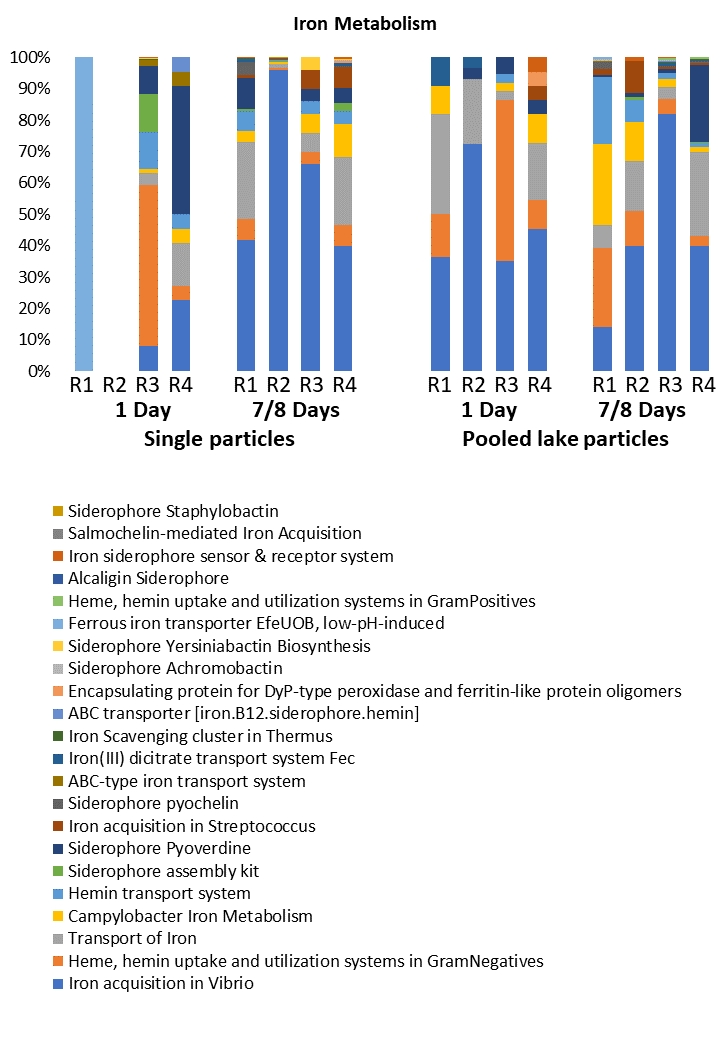

Supplement: FIGURE S5 — A subset of functional modules involved in iron metabolism and utilization obtained from the single particles and pooled lake particles transcriptomes. The shown modules are Level-3 MG-RAST subsystem hierarchy. Abundance refers to percent of reads out of total attributed to iron metabolism. The second replicate of short incubation single particles (R2) resulted in no transcripts related to iron metabolism. [file Image_5.JPEG]

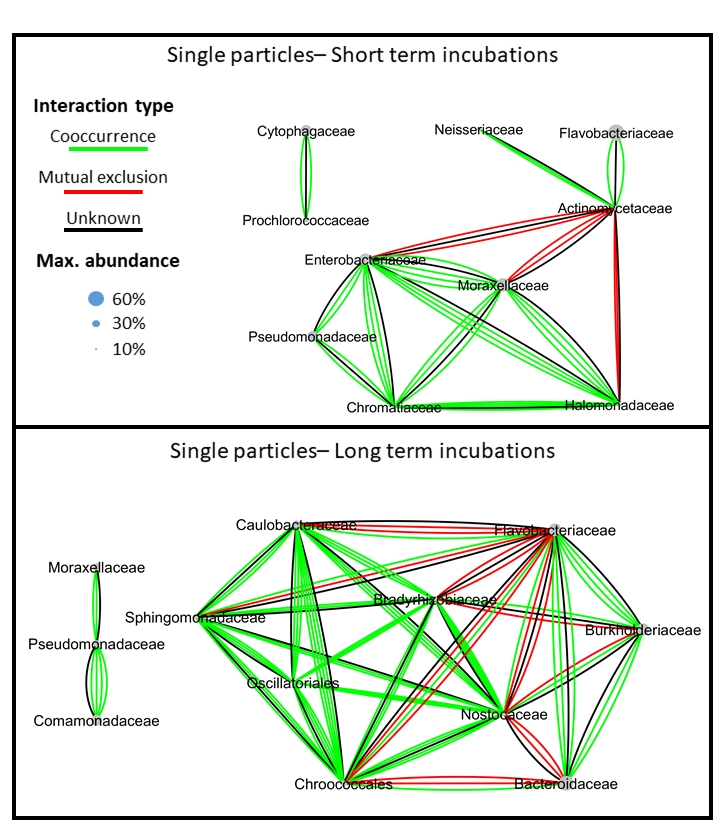

Supplement: FIGURE S6 — Co-occurrence networks of communities associated with single particles at the family level from short- and long-term incubations as calculated using CoNet (Faust and Raes, 2016). Depicted nodes are supported by at least 3 correlation or similarity methods. Node size represents maximal abundance (percent of reads attributed to taxa). Flavobacteriaceae, Moraxellaceae, and Pseudomonadaceae are the only common species between these networks, however, without common associations to other species. [file Image_6.JPEG]

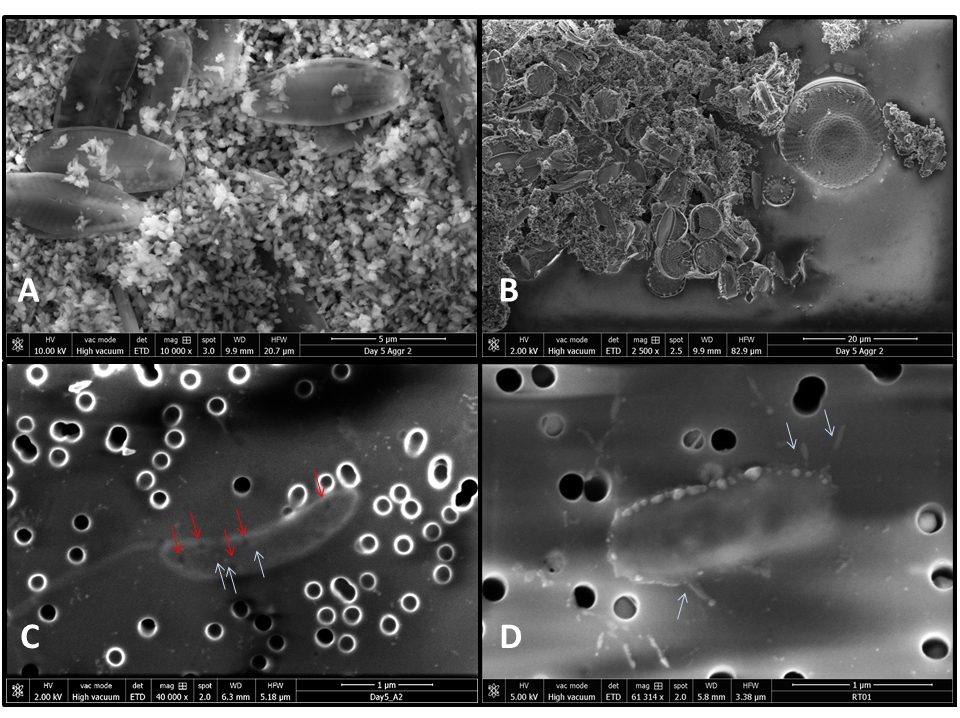

Supplement: FIGURE S7 — Scanning electron micrograph of an individual particle from Day 1 (A) or Day 5 (B). Phage-like particles were observed on cells resembling Caulobacter (C) as well as on other bacteria (D). Red and white arrows represent potential exit sites of phage particles and attached phages, respectively. [file Image_7.JPEG]
