# Supplementary material for: Organic Particles: Heterogeneous Hubs for Microbial Interactions in Aquatic Ecosystems
Source: Front Microbiol. 2018 Oct 26;9:2569. doi: 10.3389/fmicb.2018.02569 (PMC6212488; doi:10.3389/fmicb.2018.02569)
Supplement: TABLE S1 — Statistical overview of the transcriptome annotation of all individual samples using the extended Trinotate pipeline. [file Table_1.PDF]

Supplementary Table 1: Statistical overview of the transcriptome annotation of all individual samples using the extended Trinotate pipeline

Assemebley		Annotation Summary			
Contigs	Transcripts	Protein	rRNA	Not Annotated	ncRNA
39,594	39,594	18,820	-	14,409	NA

Annotation success of individual tools

BlastX		BlastP		Pfam	Prokka	KEGG	
Swiss prot	TrEMBL	Swiss prot	TrEMBL			BlastX	BlastP
10104	19483	3528	7670	4419	16282	3223	1169

CAZZY BlaxtX ($E<10^{-3}$)

AA	CE	PL	GH	GT
191	447	245	960	1,111

CAZZY BlaxtP ($E<10^{-3}$)

AA	CE	PL	GH	GT
104	177	164	410	459

Scop ($E<10^{-3}$)

Superfamily	Family
3,947	2,124
