# Supplementary material for: Organic Particles: Heterogeneous Hubs for Microbial Interactions in Aquatic Ecosystems
Source: Front Microbiol. 2018 Oct 26;9:2569. doi: 10.3389/fmicb.2018.02569 (PMC6212488; doi:10.3389/fmicb.2018.02569)
Supplement: TABLE S2 — Statistical overview of the transcriptome annotation of pooled duplicate samples using the Trinotate pipeline. [file Table_2.PDF]

Supplementary Table 2: Statistical overview of the transcriptome annotation of pooled duplicate samples using the Trinotate pipeline

<table>
<tr><th colspan="2">Assemebley</th><th colspan="4">Annotation Summary</th></tr>
<tr><th>Contigs</th><th>Transcripts</th><th>Protein</th><th>rRNA</th><th>Not Annotated</th><th>ncRNA</th></tr>
<tr><td>4,352</td><td>4,611</td><td>2,003</td><td>314</td><td>2,294</td><td>2</td></tr>
</table>

<table>
<tr><th colspan="7">Annotation success of individual tools</th></tr>
<tr><th>BlastX</th><th>BlastP</th><th>Pfam</th><th>eggnog</th><th>Kegg</th><th>MG RAST</th><th>rRNA</th></tr>
<tr><td>1,349</td><td>1,159</td><td>1,334</td><td>928</td><td>878</td><td>1,687</td><td>314</td></tr>
</table>
