# Supplementary material for: Organic Particles: Heterogeneous Hubs for Microbial Interactions in Aquatic Ecosystems
Source: Front Microbiol. 2018 Oct 26;9:2569. doi: 10.3389/fmicb.2018.02569 (PMC6212488; doi:10.3389/fmicb.2018.02569)
Supplement: TABLE S3 — List of transcripts in which CRISPR spacers were identified. Spacers location and coverage details are provided as well. Spacer sequences are given in Supplementary Data Sheet S4. [file Table_3.PDF]

#ID	Path	Avg_fold	Length	Covered_percent
TRINITY_DN274_c0_g1_i1	[481:0-86 482:87-248] [-1, 481, 482, -2]	0.0924	249	9.2369
TRINITY_DN453_c1_g1_i1	[421:0-118 422:119-214] [-1, 421, 422, -2]	1.3488	215	44.6512
TRINITY_DN453_c1_g2_i1	[423:0-136 424:137-232] [-1, 423, 424, -2]	0.2146	233	10.7296
TRINITY_DN513_c0_g1_i1	[914:0-433 915:434-438 916:439-849] [-1, 914, 915, 916, -2]	0.3082	850	24.9412
TRINITY_DN513_c0_g2_i1	[912:0-101 913:102-512] [-1, 912, 913, -2]	0.0994	513	9.9415
TRINITY_DN754_c0_g1_i1	[3473:0-36 3474:37-63 3475:64-77 3477:78-1723 3468:1724-1747 3469:1748-1758 3470:1759-1772 3471:1773-1799 3472:1800-1813] [-1, 3473, 3474, 3475, 3477, 3468, 3469, 3470, 3471, 3472, -2]	0.0347	1814	3.473
TRINITY_DN796_c0_g1_i1	[925:0-103 926:104-127 927:128-175 928:176-464] [-1, 925, 926, 927, 928, -2]	0.1806	465	16.5591
TRINITY_DN870_c0_g1_i1	[455:0-236 456:237-450] [-1, 455, 456, -2]	0.0377	451	3.7694
TRINITY_DN1067_c0_g1_i1	[6445:0-37 6449:38-247 6443:248-271 6444:272-3218] [-1, 6445, 6449, 6443, 6444, -2]	0.005	3219	0.497
TRINITY_DN1317_c0_g1_i1	[838:0-340 839:341-364 840:365-410 841:411-728] [-1, 838, 839, 840, 841, -2]	0.0316	729	3.155
TRINITY_DN1329_c0_g1_i1	[708:0-80 709:81-104 710:105-363] [-1, 708, 709, 710, -2]	0.4396	364	22.2527
TRINITY_DN1371_c0_g1_i1	[1029:0-1050] [-1, 1029, -2]	0.0152	1051	1.5224
TRINITY_DN1481_c0_g1_i1	[386:0-79 387:80-80 388:81-89 389:90-110 390:111-134 391:135-136 392:137-137 396:138-146 395:147-191 381:192-193 382:194-194 383:195-224 384:225-248 385:249-250] [-1, 386, 387, 388, 389, 390, 391, 392, 396, 395, 381, 382, 383, 384, 385, -2]	1.5857	251	82.8685
TRINITY_DN1520_c0_g1_i1	[268:0-195 269:196-228] [-1, 268, 269, -2]	0.1004	229	10.0437
TRINITY_DN1631_c0_g2_i1	[2687:0-303 2688:304-1365] [-1, 2687, 2688, -2]	0.0176	1366	1.757
TRINITY_DN1699_c0_g1_i1	[462:0-136 463:137-237 464:238-351] [-1, 462, 463, 464, -2]	0.1364	352	6.8182
TRINITY_DN1787_c0_g1_i1	[1128:0-586] [-1, 1128, -2]	0.0273	587	2.7257
TRINITY_DN1802_c1_g1_i1	[517:0-280] [-1, 517, -2]	0.0747	281	7.4733
TRINITY_DN2046_c0_g1_i1	[641:0-185 642:186-192 647:193-342 645:343-349 646:350-512] [-1, 641, 642, 647, 645, 646, -2]	0.2203	513	11.1111
TRINITY_DN2210_c0_g1_i1	[854:0-245 855:246-246 857:247-350 852:351-351 853:352-448] [-1, 854, 855, 857, 852, 853, -2]	0.3252	449	32.5167
TRINITY_DN2424_c0_g1_i1	[555:0-166 556:167-242] [-1, 555, 556, -2]	0.7695	243	44.856
TRINITY_DN2424_c0_g2_i1	[553:0-166 554:167-222] [-1, 553, 554, -2]	0.3722	223	13.0045
TRINITY_DN2440_c0_g1_i1	[215:0-236] [-1, 215, -2]	0.0802	237	8.0169
TRINITY_DN2489_c0_g1_i1	[1244:0-111 1246:112-139 1242:140-179 1243:180-516] [-1, 1244, 1246, 1242, 1243, -2]	0.1702	517	15.8607
TRINITY_DN2541_c0_g2_i1	[2179:0-159 2181:160-797 2172:798-804 2173:805-941] [-1, 2179, 2181, 2172, 2173, -2]	0.0393	942	3.9278
TRINITY_DN2582_c0_g1_i1	[2275:0-704 2276:705-723 2277:724-804] [-1, 2275, 2276, 2277, -2]	0.077	805	3.8509
TRINITY_DN2588_c0_g1_i1	[654:0-316 655:317-317 656:318-347 657:348-348 658:349-378] [-1, 654, 655, 656, 657, 658, -2]	0.7916	379	79.1557
TRINITY_DN2644_c0_g1_i1	[292:0-313] [-1, 292, -2]	0.7389	314	27.0701
TRINITY_DN2651_c0_g1_i1	[971:0-150 972:151-500] [-1, 971, 972, -2]	0.1557	501	15.5689
TRINITY_DN2709_c1_g1_i1	[880:0-406 881:407-443] [-1, 880, 881, -2]	0.0518	444	5.1802
TRINITY_DN2802_c1_g1_i1	[745:0-766] [-1, 745, -2]	0.1877	767	6.5189
TRINITY_DN2990_c0_g1_i1	[787:0-364 788:365-403] [-1, 787, 788, -2]	0.5074	404	21.0396
TRINITY_DN3043_c0_g1_i1	[655:0-349] [-1, 655, -2]	0.2	350	10
TRINITY_DN3240_c1_g1_i1	[179:0-200] [-1, 179, -2]	0.2289	201	11.4428
TRINITY_DN3333_c0_g1_i1	[4823:0-612 4824:613-616 4825:617-618 4826:619-622 4827:623-624 4828:625-628 4829:629-630 4830:631-634 4848:635-2395 4833:2396-2419 4847:2420-3009 4849:3010-3013 4850:3014-3015 4851:3016-3019 4852:3020-3021 4853:3022-3025 4854:3026-3027 4855:3028-3031 4856:3032-3033 4857:3034-3037 4822:30	0.0077	4799	0.771
TRINITY_DN3534_c0_g1_i1	[3494:0-1720 3495:1721-1744 3496:1745-3442] [-1, 3494, 3495, 3496, -2]	0.0067	3443	0.668
TRINITY_DN3676_c0_g1_i1	[1202:0-136 1203:137-151 1204:152-265] [-1, 1202, 1203, 1204, -2]	0.7218	266	12.0301
TRINITY_DN4163_c1_g1_i1	[403:0-175 404:176-223 405:224-271] [-1, 403, 404, 405, -2]	0.1691	272	10.6618
TRINITY_DN4356_c0_g1_i1	[367:0-205] [-1, 367, -2]	0.1359	206	13.5922
TRINITY_DN4421_c0_g1_i1	[1191:0-617] [-1, 1191, -2]	0.0793	618	7.9288
TRINITY_DN4436_c0_g1_i1	[463:0-253] [-1, 463, -2]	0.1575	254	15.748
TRINITY_DN4531_c0_g2_i1	[3212:0-1545 3213:1546-1600 3214:1601-3123] [-1, 3212, 3213, 3214, -2]	0.0051	3124	0.5122
TRINITY_DN4964_c0_g1_i1	[626:0-49 627:50-50 628:51-58 648:59-68 651:69-69 649:70-77 646:78-93 615:94-103 616:104-220] [-1, 626, 627, 628, 648, 651, 649, 646, 615, 616, -2]	0.8959	221	24.4344
TRINITY_DN4964_c0_g3_i1	[633:0-32 634:33-33 635:34-41 652:42-51 650:52-52 647:53-76 644:77-86 645:87-203] [-1, 633, 634, 635, 652, 650, 647, 644, 645, -2]	0.3627	204	18.1373
TRINITY_DN5072_c0_g2_i1	[1070:0-259 1071:260-271 1072:272-311] [-1, 1070, 1071, 1072, -2]	0.3526	312	24.0385
TRINITY_DN5081_c0_g1_i1	[623:0-54 624:55-256] [-1, 623, 624, -2]	0.0739	257	7.393
TRINITY_DN5258_c0_g1_i1	[1675:0-221 1676:222-243 1693:244-387 1684:388-388 1685:389-389 1686:390-390 1687:391-447 1688:448-489] [-1, 1675, 1676, 1693, 1684, 1685, 1686, 1687, 1688, -2]	0.2265	490	13.2653
TRINITY_DN5258_c0_g2_i1	[1689:0-43 1690:44-107 1691:108-108 1692:109-326] [-1, 1689, 1690, 1691, 1692, -2]	0.4954	327	49.5413
TRINITY_DN5277_c0_g1_i1	[621:0-77 622:78-101 623:102-102 624:103-118 625:119-145 626:146-171 627:172-233] [-1, 621, 622, 623, 624, 625, 626, 627, -2]	0.0726	234	7.265
TRINITY_DN5462_c0_g1_i1	[1365:0-704] [-1, 1365, -2]	0.0879	705	4.3972
TRINITY_DN5482_c0_g1_i1	[642:0-84 643:85-85 644:86-109 645:110-124 646:125-201] [-1, 642, 643, 644, 645, 646, -2]	0.2673	202	13.3663
TRINITY_DN5511_c0_g1_i1	[905:0-76 906:77-117 915:118-275 913:276-382] [-1, 905, 906, 915, 913, -2]	0.094	383	9.3995
TRINITY_DN5533_c0_g1_i1	[1285:0-664] [-1, 1285, -2]	0.1188	665	7.6692
TRINITY_DN5533_c1_g1_i1	[423:0-233] [-1, 423, -2]	0.1239	234	12.3932
TRINITY_DN5630_c0_g2_i1	[1423:0-164 1424:165-168 1425:169-227 1426:228-228 1427:229-291] [-1, 1423, 1424, 1425, 1426, 1427, -2]	0.161	292	8.2192
TRINITY_DN5638_c0_g1_i1	[730:0-194 731:195-218 732:219-232 733:233-233 734:234-250] [-1, 730, 731, 732, 733, 734, -2]	0.5657	251	14.3426
TRINITY_DN5728_c0_g3_i1	[3407:0-1010 3408:1011-1023 3423:1024-1123 3420:1124-1128 3421:1129-2116] [-1, 3407, 3408, 3423, 3420, 3421, -2]	0.0076	2117	0.7558
TRINITY_DN5815_c0_g1_i1	[2939:0-2960] [-1, 2939, -2]	0.0061	2961	0.6079
TRINITY_DN5804_c0_g1_i1	[1626:0-584 1627:585-608 1628:609-822] [-1, 1626, 1627, 1628, -2]	0.0292	823	2.9162
TRINITY_DN6107_c0_g1_i1	[3887:0-1946 3888:1947-1960] [-1, 3887, 3888, -2]	0.0092	1961	0.9179
TRINITY_DN6116_c1_g1_i1	[1013:0-207 1014:208-621] [-1, 1013, 1014, -2]	0.0772	622	3.8585
TRINITY_DN6173_c0_g2_i1	[2936:0-695 2937:696-709 2952:710-1200 2953:1201-1218 2931:1219-1235 2932:1236-1248] [-1, 2936, 2937, 2952, 2953, 2931, 2932, -2]	0.012	1249	1.201
TRINITY_DN6247_c0_g1_i1	[775:0-409] [-1, 775, -2]	0.0585	410	5.8537
TRINITY_DN6347_c0_g1_i1	[588:0-39 589:40-43 590:44-113 591:114-130 592:131-134 593:135-204] [-1, 588, 589, 590, 591, 592, 593, -2]	0.1902	205	19.0244
TRINITY_DN6347_c0_g2_i1	[594:0-96 595:97-113 596:114-117 597:118-130 598:131-207] [-1, 594, 595, 596, 597, 598, -2]	0.375	208	37.5
TRINITY_DN6417_c0_g1_i1	[857:0-61 858:62-109 867:110-295 861:296-331] [-1, 857, 858, 867, 861, -2]	0.0693	332	6.9277
TRINITY_DN6442_c2_g1_i1	[953:0-498] [-1, 953, -2]	0.0922	499	4.6092
TRINITY_DN6687_c0_g2_i1	[764:0-203 765:204-220 766:221-247] [-1, 764, 765, 766, -2]	0.1008	248	10.0806
TRINITY_DN6714_c0_g1_i1	[765:0-178 766:179-210 767:211-326] [-1, 765, 766, 767, -2]	0.0703	327	7.0336
TRINITY_DN6771_c0_g1_i1	[1882:0-167 1883:168-277] [-1, 1882, 1883, -2]	0.0863	278	8.6331
TRINITY_DN6835_c0_g1_i1	[1752:0-116 1753:117-288] [-1, 1752, 1753, -2]	0.0865	289	8.6505
TRINITY_DN6899_c0_g1_i1	[620:0-95 626:96-97 631:98-151 628:152-161 624:162-207 619:208-261] [-1, 620, 626, 631, 628, 624, 619, -2]	0.3092	262	30.1527
TRINITY_DN6899_c0_g2_i1	[608:0-74 632:75-106 629:107-116 633:117-168 630:169-181 627:182-254 623:255-284 598:285-357] [-1, 608, 632, 629, 633, 630, 627, 623, 598, -2]	0.1341	358	13.4078
TRINITY_DN6930_c0_g1_i1	[3269:0-201 3270:202-225 3275:226-1583 3273:1584-1607 3274:1608-1786] [-1, 3269, 3270, 3275, 3273, 3274, -2]	0.0145	1787	1.455
TRINITY_DN6936_c0_g1_i1	[2818:0-275 2819:276-276 2820:277-286 2849:287-517 2830:518-518 2831:519-594] [-1, 2818, 2819, 2820, 2849, 2830, 2831, -2]	0.2992	595	6.2185
TRINITY_DN6936_c0_g2_i1	[2837:0-65 2838:66-66 2851:67-369 2841:370-450] [-1, 2837, 2838, 2851, 2841, -2]	0.1286	451	12.8603
TRINITY_DN6987_c0_g2_i1	[1404:0-83 1414:84-412 1409:413-436 1410:437-453 1411:454-621] [-1, 1404, 1414, 1409, 1410, 1411, -2]	0.0386	622	3.8585
TRINITY_DN7007_c0_g1_i1	[1009:0-76 1010:77-80 1011:81-96 1012:97-112 1013:113-150 1014:151-154 1015:155-217] [-1, 1009, 1010, 1011, 1012, 1013, 1014, 1015, -2]	0.1193	218	11.9266
TRINITY_DN7049_c0_g1_i1	[703:0-50 704:51-329] [-1, 703, 704, -2]	0.1394	330	6.9697

#ID	Path	Avg_fold	Length	Covered_percent
TRINITY_DN7131_c0_g1_i1	[1869:0-790 1870:791-908] [-1, 1869, 1870, -2]	0.0231	909	2.3102
TRINITY_DN7178_c3_g1_i1	[603:0-173 612:174-321 606:322-445] [-1, 603, 612, 606]	0.0538	446	5.3812
TRINITY_DN7197_c0_g1_i1	[5076:0-1485 5095:1486-1580 5085:1581-1627 5092:1628-2073 5080:2074-2092 5084:2093-2262 5063:2263-2285 5064:2286-2309 5065:2310-2324 5088:2325-2419 5082:2420-2612 5071:2613-2635 5072:2636-2659 5073:2660-2674] [-1, 5076, 5095, 5085, 5092, 5080, 5084, 5063, 5064, 5065, 5088, 5082, 5071, 5072, 5073,	0.0093	2675	0.9346
TRINITY_DN7295_c0_g2_i1	[3291:0-214 3292:215-569] [-1, 3291, 3292, -2]	0.2	570	20
TRINITY_DN7471_c0_g1_i1	[551:0-43 552:44-92 553:93-103 565:104-116 542:117-133 543:134-137 544:138-138 545:139-162 569:163-169 568:170-181 567:182-230 570:231-241 557:242-251 558:252-275 559:276-287] [-1, 551, 552, 553, 565, 542, 543, 544, 545, 569, 568, 567, 570, 557, 558, 559, -2]	0.0521	288	5.2083
TRINITY_DN7500_c0_g1_i1	[805:0-291 806:292-370] [-1, 805, 806, -2]	0.0889	371	8.8949
TRINITY_DN7544_c0_g1_i1	[1405:0-48 1430:49-146 1436:147-218 1433:219-226 1429:227-244 1435:245-248 1432:249-266 1428:267-356 1422:357-454] [-1, 1405, 1430, 1436, 1433, 1429, 1435, 1432, 1428, 1422]	0.3033	455	22.1978
TRINITY_DN7544_c0_g1_i2	[1395:0-149 1396:150-216 1435:217-220 1432:221-238 1428:239-328] [-1, 1395, 1396, 1435, 1432, 1428]	0.1459	329	7.2948
TRINITY_DN7616_c0_g1_i1	[1987:0-290 1988:291-347] [-1, 1987, 1988, -2]	1.3362	348	27.5862
TRINITY_DN7616_c0_g2_i1	[1978:0-77 1979:78-78 1980:79-84 2003:85-140 2005:141-141 2004:142-181 2002:182-267 1983:268-291 1984:292-323 1985:324-348 1986:349-505] [-1, 1978, 1979, 1980, 2003, 2005, 2004, 2002, 1983, 1984, 1985, 1986, -2]	0.4308	506	16.996
TRINITY_DN7673_c0_g2_i1	[2961:0-382 2962:383-400 2963:401-449] [-1, 2961, 2962, 2963, -2]	0.2044	450	13.5556
TRINITY_DN7710_c0_g1_i1	[6317:0-2361 6318:2362-2385 6328:2386-2652 6310:2653-2676 6334:2677-2829 6330:2830-2886 6327:2887-2964 6332:2965-2988 6329:2989-2997 6326:2998-3021 6308:3022-5360] [-1, 6317, 6318, 6328, 6310, 6334, 6330, 6327, 6332, 6329, 6326, 6308, -2]	0.0052	5361	0.5223
TRINITY_DN7770_c1_g1_i1	[1814:0-160 1815:161-184 1816:185-209 1817:210-230 1818:231-235 1819:236-244 1820:245-272] [-1, 1814, 1815, 1816, 1817, 1818, 1819, 1820, -2]	0.1648	273	8.4249
TRINITY_DN7964_c8_g1_i1	[1533:0-788] [-1, 1533, -2]	0.0203	789	2.0279
TRINITY_DN8069_c0_g1_i1	[1245:0-544 1246:545-545 1247:546-554] [-1, 1245, 1246, 1247, -2]	0.0378	555	3.7838
TRINITY_DN8068_c1_g1_i1	[1549:0-36 1550:37-664] [-1, 1549, 1550, -2]	0.0226	665	2.2556
TRINITY_DN8081_c0_g2_i1	[3099:0-729 3113:730-757 3111:758-792 3109:793-804 3104:805-828 3067:829-1535] [-1, 3099, 3113, 3111, 3109, 3104, 3067, -2]	0.0352	1536	3.5156
TRINITY_DN8081_c1_g1_i1	[11239:0-85 11632:86-86 11610:87-87 11581:88-171 11496:172-244 11346:245-262 11419:263-266 11487:267-267 11333:268-270 11407:271-351 11117:352-352 11321:353-627 11559:628-628 11516:629-629 11448:630-643 11369:644-653 11284:654-677 11443:678-703 11364:704-746 11279:747-770 11091:771-779 110	1.0441	794	27.204
TRINITY_DN8081_c1_g1_i2	[11239:0-85 11632:86-86 11610:87-87 11581:88-171 11425:172-227 11347:228-228 11493:229-229 11415:230-230 11334:231-233 11482:234-234 11405:235-238 11322:239-247 11475:248-248 11399:249-250 11315:251-252 11469:253-256 11393:257-261 11308:262-273 11031:274-277 11032:278-278 11033:279-281 110	0.1102	363	5.5096
TRINITY_DN8081_c1_g1_i3	[11239:0-85 11632:86-86 11610:87-87 11581:88-171 11496:172-244 11345:245-271 11624:272-290 11599:291-291 11565:292-315 11524:316-316 11462:317-325 11303:326-383 11385:384-384 11460:385-387 11300:388-477 11662:478-485 11767:486-486 11786:487-487 11802:488-488 11292:489-551 11559:552-552 115	0.0696	718	3.4819
TRINITY_DN8081_c1_g1_i4	[10712:0-193 11582:194-253 11545:254-260 11498:261-273 11580:274-274 11543:275-277 11491:278-278 11413:279-282 10702:283-314 11645:315-323 11624:324-342 11599:343-343 11565:344-367 11524:368-368 11462:369-377 10735:378-383 11686:384-401 11668:402-413 11771:414-414 11789:415-416 10893:417-4	0.1091	1100	8.8182
TRINITY_DN8081_c1_g1_i6	[10835:0-58 11432:59-74 11502:75-122 11734:123-153 11718:154-154 11702:155-159 11679:160-160 11662:161-168 11767:169-169 11786:170-170 11802:171-171 11292:172-234 11559:235-235 11516:236-236 11448:237-250 11369:251-260 11284:261-284 11443:285-310 11364:311-353 11279:354-377 11091:378-386 1	0.1247	401	6.2344
TRINITY_DN8081_c1_g1_i9	[10915:0-36 10916:37-38 11430:39-90 11352:91-127 11497:128-128 11421:129-130 11342:131-132 11490:133-136 11412:137-141 11331:142-163 11679:164-164 11662:165-172 11767:173-173 11786:174-174 11802:175-175 11518:176-178 11451:179-179 11373:180-183 11768:184-192 11787:193-193 11803:194-196 117	0.5049	204	19.6078
TRINITY_DN8081_c1_g2_i3	[10865:0-27 11356:28-88 11546:89-89 11500:90-92 11427:93-173 11237:174-181 11238:182-231] [-1, 10865, 11356, 11546, 11500, 11427, 11237, 11238, -2]	0.3491	232	23.7069
TRINITY_DN8081_c2_g1_i1	[955:0-499] [-1, 955, -2]	0.124	500	6.2
TRINITY_DN8112_c2_g1_i1	[13442:0-967 13443:968-991 13448:992-3364 13433:3365-3388 13446:3389-4861 13439:4862-4885 13440:4886-4960 13449:4961-5049 13447:5050-5073 13445:5074-5465 13436:5466-5489 13437:5490-6598] [-1, 13442, 13443, 13448, 13433, 13446, 13439, 13440, 13449, 13447, 13445, 13436, 13437, -2]	0.0035	6599	0.3485
TRINITY_DN8113_c1_g1_i7	[14989:0-144 14990:145-190 15224:191-244 15154:245-247 15246:248-319 15223:320-338 15153:339-362 15310:363-367 15222:368-388 15151:389-412 15280:413-447 15266:448-486 15244:487-543 15220:544-563 15148:564-1926 15302:1927-1941 15298:1942-1947 15293:1948-1965 15286:1966-1979 15275:1980-202	0.0069	2174	0.69
TRINITY_DN8156_c3_g1_i5	[35313:0-431 35456:432-501 35399:502-525 35433:526-553 35354:554-577 35397:578-591 35431:592-615 35352:616-646 35395:647-650 35429:651-682 35349:683-703 35393:704-778 35428:779-811 35346:812-881] [-1, 35313, 35456, 35399, 35433, 35354, 35397, 35431, 35352, 35395, 35429, 35349, 35393, 35428, 3534	0.0295	882	2.9478
TRINITY_DN8186_c0_g2_i1	[14666:0-88 14991:89-102 14981:103-110 14964:111-111 14912:112-186 14938:187-285 14893:286-355 14903:356-563 14974:564-602 14956:603-693 14834:694-700 14835:701-716 14836:717-746] [-1, 14666, 14991, 14981, 14964, 14912, 14938, 14893, 14903, 14974, 14956, 14834, 14835, 14836, -2]	0.0723	747	3.6145
TRINITY_DN8186_c0_g8_i1	[14677:0-44 14983:45-55 14920:56-76 14962:77-116 15007:117-136 15011:137-141 14989:142-232 15008:233-246 14957:247-467 15012:468-472 15014:473-488 15016:489-533 14558:534-537] [-1, 14677, 14983, 14920, 14962, 15007, 15011, 14989, 15008, 14957, 15012, 15014, 15016, 14558, -2]	0.0743	538	3.7175
TRINITY_DN8190_c0_g1_i1	[3108:0-88 3109:89-101 3110:102-129 3111:130-133 3112:134-162 3195:163-239 3144:240-274 3180:275-341 3162:342-363 3206:364-417 3177:418-443 3160:444-510 3138:511-647 3029:648-648 3159:649-663 3137:664-664 3176:665-674 3205:675-682 3216:683-704 3193:705-707 3204:708-717 3175:718-741 3192:742-	0.1248	1066	8.1614
TRINITY_DN8209_c0_g1_i1	[11776:0-1827 11777:1828-1851 11778:1852-5887] [-1, 11776, 11777, 11778, -2]	0.0068	5888	0.6793
TRINITY_DN8210_c8_g4_i1	[10584:0-160 10585:161-184 10586:185-215 10587:216-218 10938:219-222 10932:223-315 10923:316-316 10901:317-337 10631:338-346 10866:347-434 10655:435-458 10656:459-467 10915:468-488 10892:489-569 10948:570-570 10807:571-593] [-1, 10584, 10585, 10586, 10587, 10938, 10932, 10923, 10901, 10631, 108	0.0286	594	2.862
TRINITY_DN8219_c0_g1_i6	[3166:0-29 3167:30-66 3719:67-78 3777:79-80 3749:81-98 3717:99-99 3680:100-137 3824:138-148 3801:149-190 3776:191-198 3747:199-203 3712:204-211 3622:212-275 4016:276-289 3745:290-316 3672:317-337 3932:338-346 3799:347-359 3902:360-403 3883:404-424 3863:425-448 3843:449-467 3820:468-501 3797:5	0.0317	789	3.1686
TRINITY_DN8303_c2_g1_i4	[5641:0-100 5735:101-115 5729:116-139 5721:140-316 5708:317-324 5727:325-375 5719:376-396 5739:397-449 5737:450-457 5726:458-508 5733:509-529 5648:530-555 5725:556-687 5740:688-711 5741:712-762 5716:763-877 5676:878-904 5677:905-954 5714:955-1589 5653:1590-1763] [-1, 5641, 5735, 5729, 5721, 570	0.0289	1764	2.8912
TRINITY_DN8303_c3_g2_i1	[3044:0-186 3165:187-216 3162:217-260 3170:261-262 3164:263-267 3169:268-277 3168:278-286 3167:287-334 3171:335-339 3163:340-395 3158:396-398 3152:399-419 3143:420-423 3132:424-447 3079:448-448 3080:449-449 3081:450-544] [-1, 3044, 3165, 3162, 3170, 3164, 3169, 3168, 3167, 3171, 3163, 3158, 3152, 3	0.6826	545	25.8716
TRINITY_DN8303_c3_g4_i1	[3031:0-71 3032:72-92 3033:93-96 3034:97-120 3035:121-121 3036:122-122 3037:123-217] [-1, 3031, 3032, 3033, 3034, 3035, 3036, 3037, -2]	0.2798	218	16.055
TRINITY_DN8352_c7_g1_i1	[2079:0-2100] [-1, 2079, -2]	0.0119	2101	1.1899
TRINITY_DN8392_c2_g1_i1	[3803:0-38 3815:39-207 3822:208-221 3817:222-222 3814:223-241 3810:242-251 3767:252-278] [-1, 3803, 3815, 3822, 3817, 3814, 3810, 3767, -2]	0.1756	279	17.5627
TRINITY_DN8392_c2_g1_i3	[3793:0-588 3821:589-625 3818:626-1066 3815:1067-1235 3822:1236-1249 3752:1250-1292 3753:1293-1318 3754:1319-1336] [-1, 3793, 3821, 3818, 3815, 3822, 3752, 3753, 3754, -2]	0.0359	1337	3.5901
TRINITY_DN8402_c4_g1_i1	[9415:0-56 9416:57-70 9417:71-103 9418:104-105 9419:106-111 9420:112-142 9421:143-143 9422:144-149 9423:150-151 9424:152-290] [-1, 9415, 9416, 9417, 9418, 9419, 9420, 9421, 9422, 9423, 9424, -2]	0.0722	291	7.2165
TRINITY_DN8448_c0_g1_i1	[1078:0-420 1079:421-450 1080:451-482 1081:483-506 1082:507-904] [-1, 1078, 1079, 1080, 1081, 1082, -2]	0.0232	905	2.3204
TRINITY_DN8699_c0_g1_i1	[1699:0-1720] [-1, 1699, -2]	0.0093	1721	0.9297
TRINITY_DN8750_c0_g1_i1	[1269:0-656 1270:657-1290] [-1, 1269, 1270, -2]	0.0356	1291	1.7816
TRINITY_DN8774_c0_g2_i1	[750:0-64 751:65-287] [-1, 750, 751, -2]	0.0833	288	8.3333
TRINITY_DN8956_c0_g1_i1	[212:0-233] [-1, 212, -2]	0.0855	234	8.547
TRINITY_DN9045_c0_g1_i1	[637:0-340 638:341-658] [-1, 637, 638, -2]	0.1806	659	12.1396
TRINITY_DN9137_c0_g1_i1	[1223:0-1244] [-1, 1223, -2]	0.0185	1245	1.8474
TRINITY_DN9356_c0_g1_i1	[698:0-719] [-1, 698, -2]	0.0403	720	4.0278
TRINITY_DN9395_c0_g1_i1	[1409:0-129 1410:130-153 1411:154-155 1419:156-233 1418:234-234 1417:235-385 1407:386-386 1416:387-541 1403:542-554] [-1, 1409, 1410, 1411, 1419, 1418, 1417, 1407, 1416, 1403, -2]	0.1802	555	9.7297
TRINITY_DN9444_c0_g1_i1	[2367:0-296 2373:297-660 2372:661-1101] [-1, 2367, 2373, 2372, -2]	0.1089	1102	7.8947
TRINITY_DN9466_c0_g1_i1	[1163:0-490 1164:491-533] [-1, 1163, 1164, -2]	0.0356	534	3.5581
TRINITY_DN9603_c0_g1_i1	[371:0-207] [-1, 371, -2]	3.0096	208	38.4615
TRINITY_DN9629_c0_g1_i1	[187:0-208] [-1, 187, -2]	2.0335	209	47.3684
TRINITY_DN10370_c0_g1_i1	[1033:0-538 1034:539-1054] [-1, 1033, 1034, -2]	0.0777	1055	3.8863
TRINITY_DN10359_c0_g1_i1	[724:0-745 725:746-1468] [-1, 724, 725, -2]	0.017	1469	1.7018
TRINITY_DN10517_c0_g1_i1	[680:0-172 681:173-196 688:197-267 687:268-337 686:338-407] [-1, 680, 681, 688, 687, 686, -2]	0.1422	408	14.2157
TRINITY_DN10866_c0_g1_i1	[375:0-209] [-1, 375, -2]	0.1238	210	12.381
TRINITY_DN10991_c0_g1_i1	[587:0-315] [-1, 587, -2]	0.0823	316	8.2278
TRINITY_DN11011_c0_g1_i1	[179:0-200] [-1, 179, -2]	0.1144	201	11.4428
TRINITY_DN11032_c0_g1_i1	[280:0-301] [-1, 280, -2]	0.053	302	5.298
TRINITY_DN11196_c0_g1_i1	[413:0-228] [-1, 413, -2]	0.3668	229	18.7773
TRINITY_DN11466_c0_g1_i1	[2891:0-1467 2892:1468-2912] [-1, 2891, 2892, -2]	0.0172	2913	0.8582
TRINITY_DN11740_c0_g1_i1	[327:0-24 340:25-161 316:162-163 317:164-164 318:165-165 319:166-167 320:168-168 321:169-169 322:170-171 323:172-172 324:173-206] [-1, 327, 340, 316, 317, 318, 319, 320, 321, 322, 323, 324, -2]	0.1208	207	12.0773
TRINITY_DN11930_c0_g1_i1	[805:0-48 806:49-284] [-1, 805, 806, -2]	0.3158	285	15.7895
TRINITY_DN11930_c0_g2_i1	[801:0-106 802:107-132 803:133-186 804:187-212] [-1, 801, 802, 803, 804, -2]	0.4883	213	24.4131
TRINITY_DN12254_c0_g1_i1	[284:0-305 285:306-588] [-1, 284, 285, -2]	0.5195	589	51.9525
TRINITY_DN12512_c0_g1_i1	[306:0-96 307:97-170 308:171-244] [-1, 306, 307, 308, -2]	0.1878	245	9.3878
TRINITY_DN12571_c0_g1_i1	[457:0-250] [-1, 457, -2]	0.1833	251	9.1633
TRINITY_DN12593_c0_g1_i1	[421:0-150 422:151-278] [-1, 421, 422, -2]	0.1935	279	19.3548
TRINITY_DN12593_c0_g2_i1	[423:0-150 424:151-156 425:157-216] [-1, 423, 424, 425, -2]	0.3594	217	17.9724
TRINITY_DN12787_c0_g1_i1	[751:0-397 752:398-772] [-1, 751, 752, -2]	0.2445	773	7.6326
TRINITY_DN12894_c0_g1_i1	[531:0-287] [-1, 531, -2]	0.309	288	22.2222
TRINITY_DN13133_c0_g1_i1	[495:0-269] [-1, 495, -2]	0.2111	270	21.1111
TRINITY_DN13142_c0_g1_i1	[887:0-455 888:456-465 889:466-475] [-1, 887, 888, 889, -2]	0.042	476	4.2017
TRINITY_DN13292_c0_g1_i1	[523:0-283] [-1, 523, -2]	0.2007	284	10.2113
TRINITY_DN13790_c0_g1_i1	[231:0-252] [-1, 231, -2]	0.4862	253	31.2253
TRINITY_DN14291_c0_g1_i1	[1027:0-1048 1028:1049-2074] [-1, 1027, 1028, -2]	0.0333	2075	3.2771
TRINITY_DN14470_c0_g1_i1	[794:0-277 795:278-278 797:279-417 792:418-418 793:419-673] [-1, 794, 795, 797, 792, 793, -2]	0.043	674	4.3027

#ID	Path	Avg_fold	Length	Covered_percent
TRINITY_DN14482_c0_g1_i1	[901:0-388 902:389-754] [-1, 901, 902, -2]	0.1272	755	12.7152
TRINITY_DN14745_c0_g1_i1	[255:0-276] [-1, 255, -2]	0.0578	277	5.7762
TRINITY_DN14920_c0_g1_i1	[793:0-418] [-1, 793, -2]	0.0573	419	5.7279
TRINITY_DN14982_c0_g1_i1	[736:0-107 750:108-264 749:265-266 748:267-376 747:377-533] [-1, 736, 750, 749, 748, 747, -2]	0.0861	534	4.3071
TRINITY_DN15332_c0_g1_i1	[262:0-283] [-1, 262, -2]	0.3099	284	30.9859
TRINITY_DN15449_c0_g1_i1	[641:0-117 642:118-157 643:158-322] [-1, 641, 642, 643, -2]	0.1207	323	12.0743
TRINITY_DN15527_c0_g1_i1	[266:0-287] [-1, 266, -2]	0.0799	288	7.9861
TRINITY_DN16091_c0_g1_i1	[443:0-243] [-1, 443, -2]	0.1148	244	11.4754
TRINITY_DN16164_c0_g1_i1	[551:0-77 552:78-296] [-1, 551, 552, -2]	0.1852	297	18.5185
TRINITY_DN16745_c0_g1_i1	[260:0-281] [-1, 260, -2]	0.1844	282	9.2199
TRINITY_DN16771_c0_g1_i1	[222:0-243] [-1, 222, -2]	0.4016	244	20.082
TRINITY_DN16793_c0_g1_i1	[483:0-263] [-1, 483, -2]	0.2235	264	22.3485
TRINITY_DN16895_c0_g1_i1	[363:0-203] [-1, 363, -2]	0.2255	204	11.2745
TRINITY_DN16922_c0_g1_i1	[198:0-219] [-1, 198, -2]	0.3409	220	23.1818
TRINITY_DN17250_c0_g1_i1	[473:0-258 474:259-494] [-1, 473, 474, -2]	0.1717	495	12.1212
TRINITY_DN17860_c0_g1_i1	[605:0-324] [-1, 605, -2]	0.2708	325	14.7692
TRINITY_DN18045_c0_g1_i1	[1423:0-733 1424:734-1444] [-1, 1423, 1424, -2]	0.063	1445	6.2976
TRINITY_DN18085_c0_g1_i1	[472:0-493] [-1, 472, -2]	0.0324	494	3.2389
TRINITY_DN18118_c0_g1_i1	[2061:0-1052] [-1, 2061, -2]	0.2944	1053	14.7198
TRINITY_DN18415_c0_g1_i1	[381:0-212] [-1, 381, -2]	0.2254	213	11.2676
TRINITY_DN18460_c0_g1_i1	[545:0-294 546:295-566] [-1, 545, 546, -2]	0.0829	567	8.2892
TRINITY_DN18557_c0_g1_i1	[356:0-377 357:378-732] [-1, 356, 357, -2]	0.5593	733	23.6016
TRINITY_DN18654_c0_g1_i1	[525:0-546] [-1, 525, -2]	0.075	547	7.4954
TRINITY_DN18661_c0_g1_i1	[232:0-253] [-1, 232, -2]	0.1969	254	9.8425
TRINITY_DN18667_c0_g1_i1	[619:0-331] [-1, 619, -2]	0.241	332	6.0241
TRINITY_DN19331_c0_g2_i1	[523:0-174 524:175-237] [-1, 523, 524, -2]	0.1891	238	18.9076
TRINITY_DN19711_c0_g1_i1	[663:0-353] [-1, 663, -2]	0.065	354	6.4972
TRINITY_DN20122_c0_g1_i1	[823:0-433] [-1, 823, -2]	0.076	434	7.6037
TRINITY_DN20790_c0_g1_i1	[337:0-358 338:359-694] [-1, 337, 338, -2]	0.1381	695	3.4532
TRINITY_DN20897_c0_g1_i1	[1171:0-607 1172:608-1192] [-1, 1171, 1172, -2]	0.0956	1193	7.544
TRINITY_DN21050_c0_g1_i1	[385:0-406] [-1, 385, -2]	0.0639	407	6.3882
TRINITY_DN21047_c0_g1_i1	[1738:0-1695 1739:1696-1696 1740:1697-1697 1741:1698-1728] [-1, 1738, 1739, 1740, 1741, -2]	0.0139	1729	1.3881
TRINITY_DN21239_c0_g1_i1	[775:0-409] [-1, 775, -2]	0.039	410	3.9024
TRINITY_DN21424_c0_g1_i1	[423:0-233] [-1, 423, -2]	0.0641	234	6.4103
TRINITY_DN21630_c0_g1_i1	[634:0-314 635:315-323 636:324-335 637:336-339 638:340-348 639:349-640] [-1, 634, 635, 636, 637, 638, 639, -2]	0.1373	641	7.4883
TRINITY_DN21709_c0_g1_i1	[182:0-203 183:204-384] [-1, 182, 183, -2]	0.0338	385	3.3766
TRINITY_DN21768_c0_g1_i1	[280:0-301] [-1, 280, -2]	0.1788	302	17.8808
TRINITY_DN22226_c0_g1_i1	[499:0-271] [-1, 499, -2]	0.0588	272	5.8824
TRINITY_DN22305_c0_g1_i1	[280:0-301] [-1, 280, -2]	0.1589	302	7.947
TRINITY_DN22361_c0_g1_i1	[211:0-232] [-1, 211, -2]	0.1073	233	10.7296
TRINITY_DN22558_c0_g1_i1	[265:0-286] [-1, 265, -2]	0.1847	287	10.8014
TRINITY_DN22821_c0_g1_i1	[185:0-206] [-1, 185, -2]	0.1111	207	11.1111
TRINITY_DN22919_c0_g1_i1	[643:0-343] [-1, 643, -2]	0.0669	344	6.686
TRINITY_DN22947_c0_g1_i1	[457:0-250] [-1, 457, -2]	0.2231	251	18.7251
TRINITY_DN23050_c0_g1_i1	[221:0-242] [-1, 221, -2]	0.0782	243	7.8189
TRINITY_DN23197_c0_g1_i1	[393:0-218] [-1, 393, -2]	0.0959	219	9.589
TRINITY_DN23249_c0_g1_i1	[673:0-358 674:359-694] [-1, 673, 674, -2]	0.0647	695	3.8849
TRINITY_DN23939_c0_g1_i1	[2205:0-1124 2206:1125-2226] [-1, 2205, 2206, -2]	0.0736	2227	4.3107
TRINITY_DN24149_c0_g1_i1	[1391:0-82 1392:83-282] [-1, 1391, 1392, -2]	0.3216	283	32.1555
TRINITY_DN24149_c0_g3_i1	[1395:0-258 1396:259-458] [-1, 1395, 1396, -2]	0.1699	459	8.4967
TRINITY_DN24442_c0_g1_i1	[391:0-217 392:218-412] [-1, 391, 392, -2]	0.0944	413	9.4431
TRINITY_DN24842_c0_g1_i1	[383:0-213] [-1, 383, -2]	0.1402	214	14.0187
TRINITY_DN24863_c0_g1_i1	[397:0-418] [-1, 397, -2]	0.1146	419	11.4558
TRINITY_DN24994_c0_g1_i1	[450:0-471] [-1, 450, -2]	0.0339	472	3.3898
TRINITY_DN25053_c0_g1_i1	[673:0-358] [-1, 673, -2]	0.1058	359	10.585
TRINITY_DN25232_c0_g1_i1	[855:0-449 856:450-876] [-1, 855, 856, -2]	0.0262	877	2.6226
TRINITY_DN25234_c0_g1_i1	[376:0-397] [-1, 376, -2]	0.2035	398	13.8191
TRINITY_DN25338_c0_g1_i1	[523:0-283] [-1, 523, -2]	0.4683	284	37.3239
TRINITY_DN25431_c0_g1_i1	[787:0-415] [-1, 787, -2]	0.125	416	6.25
TRINITY_DN26128_c0_g1_i1	[190:0-211 191:212-400] [-1, 190, 191, -2]	0.0748	401	7.4813
TRINITY_DN26308_c0_g1_i1	[194:0-215] [-1, 194, -2]	0.1065	216	10.6481
TRINITY_DN26335_c0_g1_i1	[437:0-240] [-1, 437, -2]	0.1992	241	9.9585
TRINITY_DN26675_c0_g1_i1	[308:0-329] [-1, 308, -2]	0.0788	330	7.8788
TRINITY_DN26688_c0_g1_i1	[179:0-200] [-1, 179, -2]	0.2687	201	13.4328
