# Supplementary material for: Organic Particles: Heterogeneous Hubs for Microbial Interactions in Aquatic Ecosystems
Source: Front Microbiol. 2018 Oct 26;9:2569. doi: 10.3389/fmicb.2018.02569 (PMC6212488; doi:10.3389/fmicb.2018.02569)
Supplement: TABLE S4 — Transcripts containing matches to polyketide synthethases and non-ribosomal peptide synthetases. [file Table_4.PDF]

Supplemetary Table 4: Transcripts containing matches to polyketide synthethases and non-ribosomal peptide synthetases.

Transcript	Match length	Annotation	E-value
TRINITY_DN3567_c0_g1_i1	524	PKS type I iterative domain KS Polyketide synthase	206.4 1E-060
TRINITY_DN16049_c0_g1_i1	64	PKS type I iterative domain AT	119.3 3E-034
TRINITY_DN20188_c0_g1_i1	21	NRPS domain A non ribosomal peptide synthase	107.8 7.1E-031
TRINITY_DN21571_c0_g1_i1	20	NRPS domain A non ribosomal peptide synthase	86.7 1.8E-024
TRINITY_DN24159_c0_g1_i1	26	NRPS domain A non ribosomal peptide synthase	81.4 7.1E-023
TRINITY_DN10711_c0_g1_i1	61	PKS type II KS beta domain	83 2.7E-023
TRINITY_DN22620_c0_g1_i1	26	NRPS domain C	71.4 8.9E-020
TRINITY_DN6044_c0_g1_i1	377	PKS type II KS beta domain	54.8 9.7E-015
TRINITY_DN15127_c0_g1_i1	37	NRPS domain A non ribosomal peptide synthase	71.8 6E-020
